# Single-cell epigenetic and transcriptomic states across the continuum of monoclonal B cell lymphocytosis to chronic lymphocytic leukemia

**DOI:** 10.1186/s13059-026-04072-4

**Published:** 2026-04-15

**Authors:** Anja C. Rathgeber, Stacey M. Fernandes, Adi Nagler, Shuqiang Li, David M. Dorfman, Lars Bullinger, Matthew S. Davids, Jennifer R. Brown, Kenneth J. Livak, Leif S. Ludwig, Catherine J. Wu, Livius Penter

**Affiliations:** 1 Berlin Institute of Health at Charité - Universitätsmedizin Berlin, Berlin, Germany; 2 Max-Delbrück-Center for Molecular Medicine in the Helmholtz Association (MDC), Berlin Institute for Medical Systems Biology (BIMSB), Berlin, Germany; 3 Department of Biology, Chemistry, Pharmacy, Freie Universität Berlin, Berlin, Germany; 4 Department of Medical Oncology, Dana-Farber Cancer Institute, Boston, MA USA; 5 Translational Immunogenomics Lab, Dana-Farber Cancer Institute, Boston, MA USA; 6 Department of Pathology, Brigham and Women’s Hospital and Harvard Medical School, Boston, MA USA; 7 Department of Hematology, Oncology, and Cancer Immunology, Berlin, Charité - Universitätsmedizin Berlin, corporate member of Freie Universität Berlin and Humboldt-Universität zu Berlin, Augustenburger Platz 1, Berlin, 13353 Germany; 8 German Cancer Consortium (DKTK), partner site Berlin, and German Cancer Research Center (DKFZ), Heidelberg, Germany; 9 National Center for Tumor Diseases (NCT), partner site Berlin, Berlin, Germany; 10 Broad Institute of Massachusetts Institute of Technology and Harvard University, Cambridge, USA; 11 Harvard Medical School, Boston, USA; 12 Berlin Institute of Health at Charité – Universitätsmedizin Berlin, BIH Biomedical Innovation Academy, BIH Charité Digital Clinician Scientist Program, Berlin, Germany

**Keywords:** Monoclonal B cell lymphocytosis, Chronic lymphocytic leukemia, Mitochondrial DNA mutations, Lineage-tracing, Multi-omics

## Abstract

**Background:**

Chronic lymphocytic leukemia (CLL) develops from physiologic B cells through low- and high-count monoclonal B cell lymphocytosis (LC-/HC-MBL). The timing and nature of early B cell expansion and molecular evolution remain unclear, limiting prediction of progression.

**Results:**

Using multi-omics single-cell sequencing integrating chromatin accessibility, transcriptional, proteomic, and mitochondrial DNA (mtDNA) profiles across normal B cells, LC-/HC-MBL, and CLL, we delineate clonal relationships and evolutionary trajectories. Our data reveals subclonal, epigenetic, and transcriptomic stability during the transition from HC-MBL to CLL, suggesting a continuous disease spectrum rather than distinct evolutionary phases. CLL-like molecular states already exist in LC-MBL and, along with individual-specific heterogeneity across HC-MBL/CLL, are linked with disease progression. Finally, we find genetic evidence for a shared progenitor between physiologic and monoclonal B cells.

**Conclusions:**

These results position LC-MBL as a key inflection point in early CLL pathogenesis and a potential target for progression risk prediction or preventive strategies.

**Supplementary Information:**

The online version contains supplementary material available at 10.1186/s13059-026-04072-4.

## Background

Chronic lymphocytic leukemia (CLL) [1] is the result of a multi-year transformation process [2] that leads to uncontrolled proliferation of mature monoclonal B cells. CLL is typically preceded by the premalignant states of initially low-count (LC-) and subsequent high-count monoclonal B cell lymphocytosis (HC-MBL) that are defined as < 500 or 500–5,000 light chain-restricted CD19^+^ CD5^+ ^cells per µl blood. LC- or HC-MBL status does not impact long-term survival, but is associated with increase in the risk for lymphoid malignancies by 4.3- and 74-fold, including progression to CLL, which takes place at a rate of approximately 0.5%/year [3, 4].

The long natural disease history and the ease of repeatedly sampling circulating tumors cells make CLL and its precursors a model for the study of indolent B cell lymphoma biology and clonal evolution. Further, deep proteogenomic characterizations of CLL have driven many therapeutic innovations for B cell lymphomas, including BTK, BCL-2 or PI3K inhibition and CD19/CD20-directed immunotherapy [5–7]. These have largely replaced traditional chemotherapies as first line treatment for CLL and dramatically improved CLL long-term outcomes [8, 9]. However, a remaining unresolved challenge in CLL clinical care is the possibility to predict or even prevent progression from MBL. Although lack of IGHV mutational status is recognized as a major risk factor in CLL, it is less frequent in MBL and thus only of limited utility for clinical risk assessment. Certainly, a better understanding of the premalignant states preceding CLL, which have been so far incompletely characterized, would lay the foundation for addressing this knowledge gap. Next-generation sequencing and functional studies have characterized recurrent hotspot mutations in genes like *SF3B1* [10], *NOTCH1*, *IKZF3* or *RPS15* [11] that initiate transformation of physiologic B cells and established that MBL and CLL share many of these genetic [4, 12–14] but also epigenetic [15, 16] features. For example, CLL-defining cytogenetic abnormalities like *del*(13q) or *tri*(12*)* are already detectable in LC-MBL [17] and the transition from HC-MBL to CLL is characterized by stable methylomes [15]. However, the transcriptional and epigenetic cell states in LC-MBL are largely unexplored and it remains unknown whether transcriptional changes or outgrowth of subclones drive the MBL/CLL transition.

Multimodal single-cell genomics approaches that co-capture natural barcodes and cell states offer an opportunity to interrogate the clonal and phenotypic relationships between physiologic and transformed B cells across the MBL/CLL continuum [18]. Copy number changes and recurrent driver mutations can identify CLL clones in single cell datasets [19]. In addition, mitochondrial DNA (mtDNA) mutations have gained traction as a tool for tracking clonal ancestries due to their unique genetics—high copy number per cell, a ten times higher mutation rate compared to nuclear DNA and a small genomic size of 16.6 kB that permits high single-cell sequencing coverage. As the heteroplasmy of mtDNA mutations has a wide range and is more dynamic than variant allele frequencies of somatic mutations due to stochastic segregation during cell division, they have the potential to resolve subtle population shifts and can improve identification of cancer subclones [20]. Approaches like the mitochondrial single cell assay for transposase-accessible chromatin using sequencing (mtscATAC-seq) have enabled longitudinal studies in blood malignancies, which confirm that changes in mtDNA mutations mirror disease history and may enable dissection of additional clonal heterogeneity alongside recurrent somatic driver mutations or copy number changes [20].

Taking advantage of these technological advances, we herein performed an integrated single cell genomics analysis of transcriptional, epigenetic and genetic profiles across the spectrum from physiologic B cells, LC- and HC-MBL to CLL. We found subclonal, epigenetic and transcriptomic stability across the HC-MBL/CLL transition, consistent with cell proliferation rather than clonal selection as driving force. Further, we reveal the presence of heterogeneous CLL-like cell states already in LC-MBL. Together, these findings suggest LC-MBL to be a decisive stage for defining the early steps of CLL-initiation.

## Results

### Generation of a single cell map spanning physiologic B cells, MBL and CLL

**Fig. 1:**
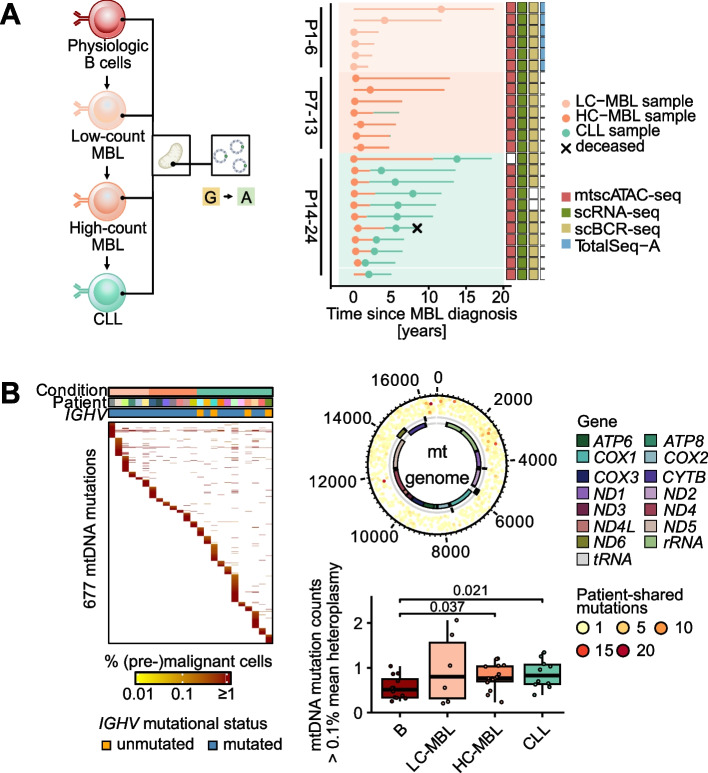
Study overview for investigating the transition from monoclonal B cell lymphocytosis to chronic lymphocytic leukemia. **A** From a healthy pool of polyclonal B cells, a monoclonal, premalignant low-count (LC)-MBL arises and evolves into high-count (HC)-MBL, before transitioning into CLL. At each stage, mtDNA mutations and B cell receptor sequences were profiled to trace the (sub-)clonal dynamics of this transition (left). Swimmers plot showing time of diagnosis and sampling for each patient (right) with profiled single-cell data modalities indicated. **B** Detected mtDNA mutations across all cell types of 34 samples (left). Recurrent mtDNA mutations across patients and genomic locations (top right). Number of mtDNA mutations > 1% mean heteroplasmy across cell types (bottom right). Samples were down-sampled to 100 cells per patient and cell type and normalized for mtDNA sequencing depth. Statistical testing using *Wilcoxon rank-sum test*

To profile the epigenetic and transcriptional evolution from physiologic B cells, LC- and HC-MBL to CLL, we obtained 301,853 scRNA- and 168,533 mtscATAC-seq profiles from CD19^+^ enriched peripheral blood samples from 24 individuals with LC- (P1-P6; *n* = 6), HC-MBL (P7-P13; *n* = 7) and CLL (P14-P24; *n* = 11) (Table 1, Additional file 2: Table S1, Additional file 1: Fig. S1A). For 10 HC-MBL cases, we included a matched CLL sample with a median follow-up of 1,926 days (range 371–5,037 days), enabling assessment of longitudinal dynamics within individuals (Fig. 1A). In the case of LC-MBL, we augmented mtscATAC-seq by capturing surface marker expression using Total-seq A (ATAC with select antigen profiling by sequencing, ASAP-seq). In addition, we acquired 236,009 single cell BCR and 148,395 mtDNA mutational profiles to enable tracking of MBL/CLL clones. In total, we found 677 mtDNA mutations (mean 38 mutations per case, range 12–120) evenly distributed across the entire mitochondrial chromosome (Fig. 1B, Additional file 1: Fig. S1B). While 567 (84%) mutations were non-recurrent, select mutations such as 16390G>A, 11711G>A or 3244G>A were found in up to 20 individuals (Additional file 2: Tables S2-3). The normalized number of identified mtDNA mutations in MBL/CLL cells was higher than in physiologic B cells (*p* < 0.05), supporting the utility of this barcode for establishing clonal relationships (Fig. 1B). The apparently lower number of mtDNA mutations in non-B compartments was likely due to fewer sequenced cells (Additional file 1: Fig. S1C-D).

**Table 1:** Clinical information of MBL/CLL single cell genomics study cohort

Cohort	Case	Age at diagnosis	Sex	IGHV	Cytogenetics	Mutated genes
LC-MBL	1	67	male	unknown	-	-
	2	68	female	unknown	-	-
	3	56	female	unknown	-	-
	4	70	female	unknown	-	-
	5	70	female	mutated	*tri(12)*	-
	6	61	male	unknown	-	-
HC-MBL	7	66	male	mutated	*del*(13q)	-
	8	53	male	mutated	*del*(13q)	-
	9	68	male	mutated	-	-
	10	60	male	mutated	*del*(13q)	-
	11	64	male	mutated	*del*(13q)	-
	12	64	female	mutated	*del*(13q), -X	*SF3B1*^*Y623C*^
	13	64	male	mutated	*del*(13q)	*DNMT3A*^W313*^, *DNMT3A*^*R676Q*^, *TET2*^*R1261G*^
HC-MBL/ CLL	14	59	male	unmutated*	*tri*(12)	-
	15	60	male	mutated*	-	-
	16	67	male	unmutated	-	*SF3B1*^*K666M*^, *TET2*^*T946fs*^, *ASXL1*^*N884fs*^, *ATM* gain
	17	65	male	mutated	*del*(13q)	-
	18	58	male	mutated	*del*(13q)	-
	19	65	female	mutated	*del*(13q)	*NOTCH1*^P2514Rfs*4^
	20	65	male	mutated	*del*(13q)	*DNMT3A*^R598*^, *TET2*^*H1382Y*^
	21	62	female	unmutated	*tri*(12)	*NOTCH1*^P2514Rfs*4^
	22	55	male	mutated	*del*(13q)	-
	23	73	male	mutated	*del*(13q), *del*(17p)	*DNMT3A*^*P904L*^, *TP53*^*R209fs*^
	24	66	male	unmutated	*del*(6q)	*NOTCH1*^P2514Rfs*4^

^*^IGHV status inferred from scBCR-seq data (Supplemental Methods)

**Fig. 2:**
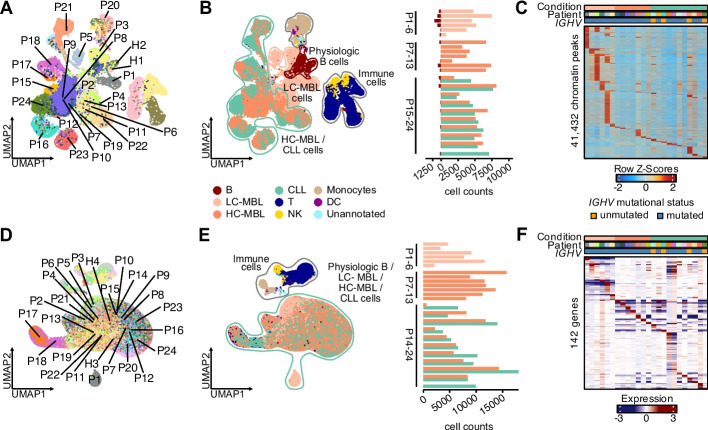
Single cell chromatin accessibility and gene expression profiles across patients and cell types. **A** UMAP of chromatin accessibility profiling with scATAC-seq (left) across 23 patients (P1-13, P15-P24) and two healthy controls (H1-H2). **B** Cell type annotation via UMAP of chromatin accessibility profiling (left) with bar plots indicating the number of physiologic B cells and (pre)malignant MBL/CLL cells per patient (right). **C** Differential MBL/CLL-associated accessible chromatin peaks across individuals. **D** UMAP of gene expression with scRNA-seq across 24 patients (P1-P24) and two healthy controls (H3-H4). **E** Malignant cell type annotation via UMAP of gene expression with bar plots indicating the number of (pre-)malignant MBL/CLL cells per patient (right). **F** Differential MBL/CLL-associated gene expression across individuals

Based on marker gene expression or transcription factor motif activity and through integration with healthy-donor PBMCs, we annotated the single cell profiles as part of the B cell compartment or as non-B immune cells, including T/NK cells, monocytes and dendritic cells (Fig. 2A-F, Additional file 1: Figs. S2A-C, S3A-C). Within the B cell compartment, we could distinguish physiologic from (pre)malignant B cells based on their chromatin accessibility, including in genes like *CD5*, *MME* (encoding CD10) and *FCER2* (CD23) (Additional file 1: Fig. S4A). Physiologic B cells represented 10.6% of B cells in LC-, but only 2.7% in HC-MBL and 0.6% in CLL. Analysis of transcriptional profiles alone, however, did not permit the distinction of physiologic from monoclonal B cells, a known limitation of CLL scRNA-seq studies, which we overcame by incorporating BCR information into subsequent analyses [15, 21, 22].

Within the physiologic B cell compartment we identified naïve, intermediate, memory B and plasma cells and found an expansion of memory B cells from LC-MBL to CLL (*p* < 0.05; *Student’s t-test*) (Additional file 1: Fig. S5A-E). Although underpowered to assess immune cell populations systematically, we nevertheless found increases of CD4^+^ and CD8^+^ effector memory T cells in peripheral blood of HC-MBL/CLL compared to healthy donors or individuals with LC-MBL (Additional file 1: Fig. S6A-E), consistent with prior reports of immune dysfunction in early stages of CLL [22].

In line with the natural disease history of CLL, physiologic B cells were more similar to LC- compared to HC-MBL or CLL cells based on chromatin accessibility (Fig. 2B, Additional file 1: Fig. S7A-left). This similarity was highest in cases with the least expansion of CD19^+^ CD5^+^ cells (Additional file 1: Fig. S7A-right). Across LC-, HC-MBL and CLL, independent of physiologic B cells, we noticed case-specific cell clusters driven by 41,432 differentially accessible chromatin peaks (out of 135,514 total peaks) associated with single individuals. This is consistent with a high degree of cell state heterogeneity within the (pre)malignant B cell compartment, which was not related to IGHV status or other clinical features (Fig. 2C, Additional file 1: Figs. S2C, 3C). This heterogeneity has been observed in other studies of CLL biology [15, 21] and suggests that beyond shared changes in CLL, individual cases acquire unique cell states, although they may also represent a certain degree of technical variability. Transcriptional diversity was more limited, with only 142 differentially expressed genes between patients detectable across the cohort, likely because individual genes contain multiple differential chromatin peaks or indirect effects of chromatin accessibility on transcriptomes (Fig. 2F).

Given the distinct chromatin accessibility profiles of physiologic and transformed B cells, we asked whether this would be reflected at the level of genetic or surface markers. Analysis of 154 surface markers in LC-MBL revealed CLL-associated expression changes of CD5 or CD200 (higher in LC-MBL) and CD49d or Integrin β7 (higher in B cells) [23]. We also detected physiologic B cells to consistently have higher expression of the 5’-nucleotidase CD73 and the complement receptor CD35, potential markers for diagnosis of CLL [24] (Additional file 1: Fig. S7B-F). Two copy number variant changes were identified in two LC-MBL cases (*del*(1p*)* and *del*(14q) in P1 and *tri*(12) in P5) (Additional file 1: Fig. S8A-B), supporting the notion of early acquisition of genetic changes in LC-MBL.

Together, these results reveal that LC-MBL is characterized by heterogeneous, individual-specific genetic, epigenetic and transcriptional states distinct from physiologic B cells that are present even in the earliest stages of CLL development.

### Genetic stability during the HC-MBL/CLL transition

**Fig. 3:**
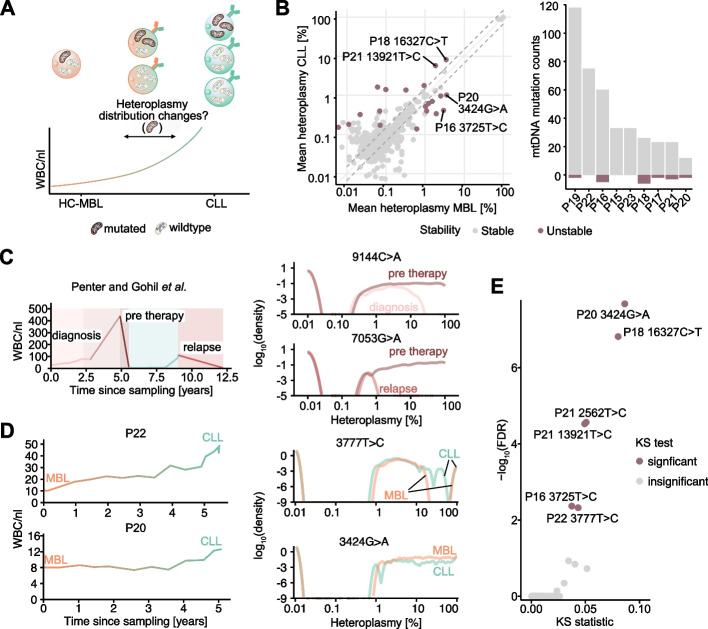
Heteroplasmy distributions during HC-MBL to CLL progression. **A** Schematic of mtDNA mutation heteroplasmy changes during MBL/CLL progression. **B** Mean heteroplasmy of mtDNA mutations for serial HC-MBL/CLL samples. Dashed lines represent the filtering threshold of absolute 1.5-fold change. *Wilcoxon rank-sum test and Benjamini–Hochberg correction*. Labeled mtDNA mutations display significantly different heteroplasmy distributions assessed via a *Kolmogorov–Smirnov test and Benjamini–Hochberg correction (FDR* ≤ *0.05)* (left). Quantification of mtDNA mutation stability based on significance filters from the left scatterplot (right). **C** Exemplary heteroplasmy distribution comparison of CLL1 from Penter and Gohil et al*.*^*15*^. White blood cell (WBC) counts are depicted (left) as a reference for the disease course. Heteroplasmy distributions of mutations 9144C>A (top) and 7053G>A (bottom) comparing diagnosis to the pre-therapy or pre-therapy to relapse timepoint. **D** WBC counts depicted for P22 and P20 (left). Heteroplasmy distributions of mtDNA mutations 3777T>C of P22 (top right) and 3424G>A of P20 (bottom right). **E** Detection of changes in mtDNA mutation distributions. *Kolmogorov–Smirnov (KS) test* and *Benjamini–Hochberg FDR* ≤ *0.05*

To determine whether MBL progresses to CLL through genetic evolution or tumor cell proliferation, we evaluated if there was evidence of leukemia evolution – through comparing the mtDNA profiles of HC-MBL with matched CLL samples (Fig. 3A). Our previous work showed that changes in mtDNA heteroplasmy closely aligned with clonal evolution that was identified through somatic mutation analysis [20, 21, 25]. We therefore analyzed longitudinal changes in our current cohort through the use of mtDNA mutations.

When comparing the mtDNA mutational profiles of MBL with matched CLL, we observed a high degree of stability. We only identified statistically significant changes of integrated mean heteroplasmies in 20 of 423 (4.73%) mtDNA mutations (absolute fold change ≥ 1.5) between matched MBL and CLL samples without clear relation to changes in circulating leukemia burden or time between sampling timepoints (Fig. 3B, Additional file 1: Fig. S9A).

This prompted us to ask whether more subtle clonal shifts between MBL and CLL are discernable through analyzing the distribution of single-cell mtDNA mutation heteroplasmies, as these can have a wide range from < 1 to 100%. As an example, we reanalyzed published mtDNA mutational profiles from a serially sampled CLL case (diagnosis, prior to immunochemotherapy and relapse) with known clonal replacement [21] and found clearly shifted heteroplasmies (Fig. 3C). To quantify changes in heteroplasmy skewing of individual mtDNA mutations between MBL and CLL, we compared their distributions in quantile–quantile analysis (“Q-Q plots”). In this analysis, a mtDNA mutation that skews towards higher or lower heteroplasmy values is identifiable as a shift in its distribution and thus a deviation from the x = y line in the Q-Q plot (Additional file 1: Fig. S9B). By applying this approach to all MBL/CLL pairs, we sensitively identified individual mtDNA mutations with subtle shifts (e.g. 3777 T>C—skewing towards higher heteroplasmies—or 3424G>A—lower heteroplasmies—in P22 and P20, respectively) (Fig. 3D). However, overall, merely 6 of 355 mtDNA mutations significantly shifted their heteroplasmy distribution (requiring ≥ 100 cells in either the MBL or CLL sample for Kolmogorov–Smirnov testing, followed by Benjamini–Hochberg correction; adjusted *p*-value ≤ 0.05), further confirming the genetic stability of the MBL/CLL transition (Fig. 3E, Additional file 1: Fig. S9C-D). These findings altogether lead us to conclude that progression from HC-MBL to CLL is most likely driven by cell proliferation rather than by clonal selection.

### CLL-like phenotypes in low-count MBL

**Fig. 4:**
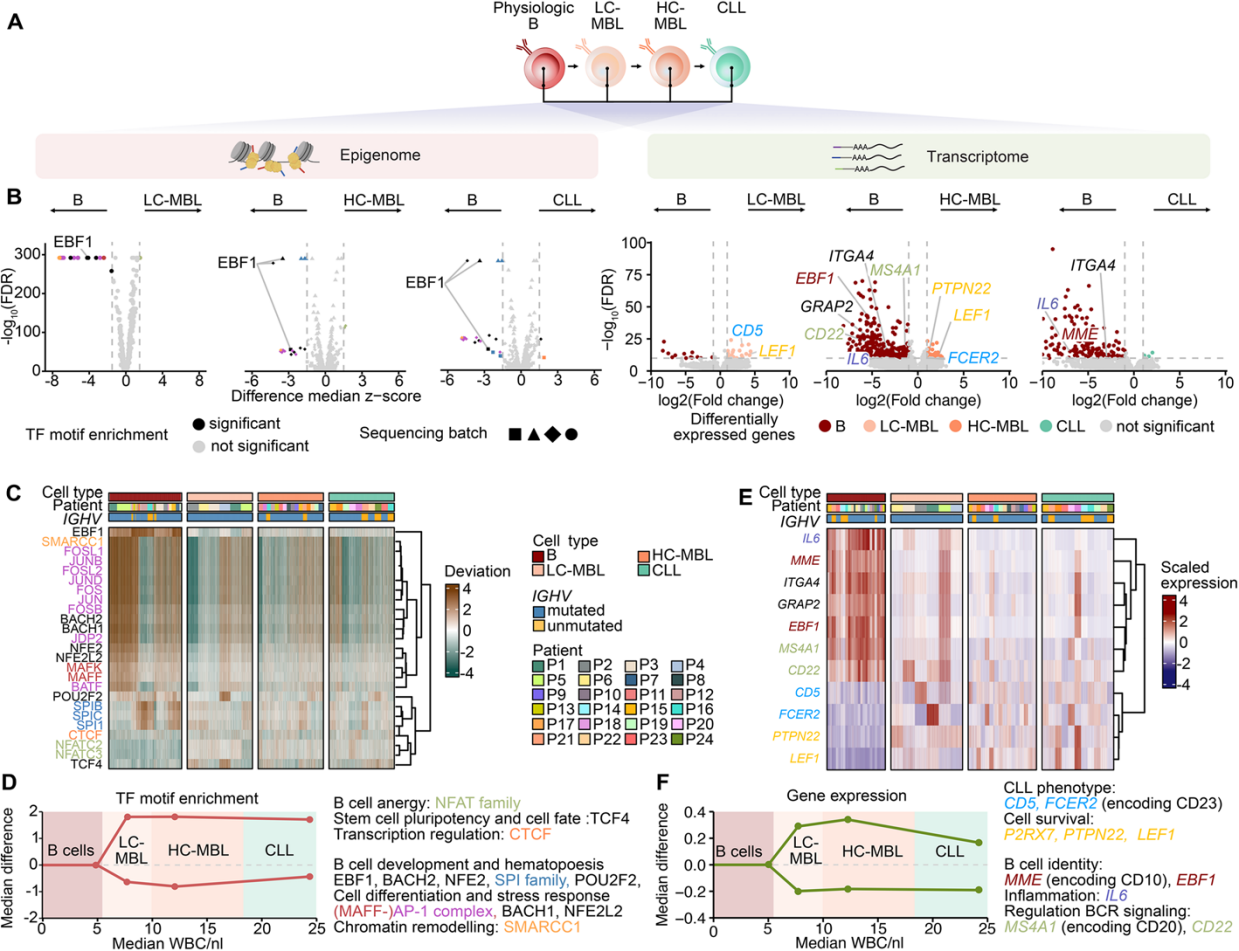
Multimodal differential analysis of MBL to CLL progression. **A** Schematic of epigenetic and transcriptomic comparative analysis between physiologic B cells, LC-MBL, HC-MBL, and CLL cells. **B** Pseudo bulk TF motif enrichments across all samples per condition. Z-scores are based on peaks from scATAC-seq data. *Wilcoxon rank-sum test (Benjamini–Hochberg correction FDR* ≤ *0.05).* Thresholds as dashed lines with an absolute difference of median z-scores > 1.5 and a cut-off at log_10_(FDR) > 10. Colored labels indicate the TF motifs belong to similar protein families or complexes. **C** Heatmap of deviation scores for all identified TF motifs from B. Individual deviation scores were equally downsampled across conditions. Deviation-score values were capped to the 93rd percentile for better visualization. TF motif names are colored by protein complexes and families. **D** Schematic of median TF motif enrichment changes across all up- or downregulated factors relative to the physiologic B cells in disease progression and CLL-associated pathways. **E** Pseudo bulk differential gene expression between physiologic B cells and LC-MBL, HC-MBL, and CLL cells across all patients per condition. Only non-immunoglobulin genes are labeled. DEGs were identified using a *Wald test* and thresholds are depicted as dashed lines with an absolute average log_2_ fold change > 1 and -log_10_(FDR) cut-off > 10. **F** Heatmap of scaled expression values for all significant DEGs from E. Expression depicted for down-sampled cells across conditions. Expression values were capped at ± 3 for better visualization. **G** Schematic showing gene expression changes across all up or downregulated genes relative to the physiologic B cells in disease progression and CLL-associated pathways

Having observed genetic stability in the HC-MBL/CLL transition with individual-specific chromatin accessibility and gene expression profiles already detectable in LC-MBL, we wondered what unifying cell state changes might drive CLL development. We thus systematically compared the single cell profiles of physiologic B cells versus LC-, HC-MBL and CLL at the epigenetic and transcriptional levels (Fig. 4A).

To detect alterations in gene regulation, we performed differential transcription factor (TF) motif enrichment analysis, which revealed more common differences between physiologic B cells and LC-MBL than between LC-MBL and HC-MBL or CLL. This analysis showed MBL/CLL to have reduced activity of TF motifs implicated in B cell development like EBF-1 [26], BACH1/2 [27], POU2F2 [28] and increased motif activity of TFs relevant to lymphomagenesis like TCF4 [29, 30] or NFAT [31] (Fig. 4B-left, Additional file 1: Fig. S10A). Such changes were already present at time of LC-MBL, and between HC-MBL and CLL no further common epigenetic changes were found (Fig. 4C-D, Additional file 1: Fig. S10B). TF activity inferred from scRNA-seq data showed similar results for 11 transcription factors compared to the ATAC-seq analysis, validating a link between changes at the level of chromatin accessibility and gene expression (Additional file 1: Fig. S11A).

Transcriptional changes across MBL/CLL mirrored these dynamics, with downregulation of B cell-associated genes such as *EBF1*, *IL6*, *MS4A1* (CD20), *MME* (CD10) or *ITGA4* (CD49d [32]) and upregulation of CLL-associated genes including *CD5*, *FCER2* (CD23), *BCL2*, *LEF1*, or *P2RX7* [33].

Interestingly, in a reanalysis of *CLL-map* bulk RNA-seq data [11], expression of *ITGA4* (encoding CD49d) was associated with IGHV unmutated status, consistent with adverse prognostic risk. Further, higher levels of *FCER2* (CD23) and *LEF1* or *MS4A1* (CD20) were associated with improved treatment failure-free survival in IGHV mutated or unmutated CLL, respectively (Additional file 1: Fig. S12A-C).

Gene enrichment analysis further supported these observations as it showed the most enriched gene set in LC-MBL to be a published CLL gene list [34] (Fig. 4B-right, E, Additional file 1: Fig. S13A). On the other hand, transcriptional differences between HC-MBL and CLL were minimal, consistent with the observed epigenetic stability (Additional file 1: Fig. S13B).

Together, these findings support a model wherein LC-MBL already acquires core CLL-like epigenetic and transcriptional programs (Fig. 4F). Progression to HC-MBL/CLL associates with acquisition of further individual chromatin accessibility and transcriptomic changes.

### Clonal relationships of physiologic and monoclonal B cells

**Fig. 5:**
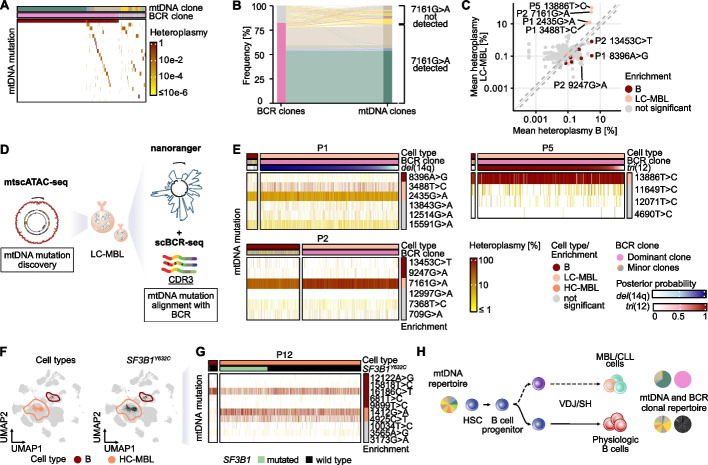
Clonal relationships between residual physiologic B cells and LC-MBL cells. **A** Simultaneous mtDNA mutation-based clones and BCR clone calling from scRNA-seq data. Heteroplasmy heatmap of patient P2 (*n* = 1,347 cells) from gene expression data. mtDNA mutation-based clones are indicated by a color spectrum and BCR sequence-based clones are colored by the major clone (pink) and all minor clones (gray). Heatmap coloring indicates mtDNA mutation heteroplasmy. **B** Alluvial plot visualizing mtDNA mutation clones’ matching with BCR sequence clones. **C** Significant heteroplasmy changes between physiologic B cells and LC-MBL, highlighted for three exemplary patients. *Wilcoxon rank-sum test and Benjamini–Hochberg FDR* ≤ *0.05.* Dashed lines indicate absolute threshold of 1.5-fold change. **D** Schematic for combined long-read sequencing read-out of BCR CDR3 clones in LC-MBLs with mtDNA mutations discovered by scATAC-seq. **E** Heatmaps of mtDNA mutation heteroplasmy (*nanoranger*) annotated with BCR clones and chromosomal aberrations identified from scRNA-seq data. **F** UMAPs of cell types and *SF3B1* mutation status in P12. **G** Heteroplasmy heatmap of mtDNA mutations (mtscATAC-seq) with significantly different heteroplasmy between physiologic B cells and HC-MBL cells in P12 and cooccurrence of *SF3B1*^*Y632C*^. **H** Model of clonal relationships based on mtDNA mutations and BCR clones under physiological conditions and in MBL/CLL

The cell-of-origin in CLL remains ill-defined, including how polyclonal B cells relate to MBL/CLL phylogenetically and whether they have a common hematopoietic progenitor. To query whether BCR and mtDNA mutational profiles of our cohort might provide orthogonal insights into the evolution of B cell subclones, we took advantage of one index case (P2) with high mitochondrial gene expression levels (Additional file 1: Fig. S14A). This permitted the combined read-out of mtDNA mutations and BCR from the same cell. In this case, the MBL-associated BCR sequence aligned almost perfectly with the 7161G>A mutation (Fig. 5A), but additional mtDNA mutations enabled the further subpartitioning of the MBL population. These included both mtDNA mutations enriched in the MBL population and those that were shared with polyclonal non-MBL B cells (Fig. 5B). To investigate these observations systematically, we compared mtDNA mutation heteroplasmies between residual matched physiologic and monoclonal B cells in 6 LC-MBL cases, where both populations were sufficiently represented. While 245 of 253 (97%) mutations were shared, 5 were selectively enriched (i.e., 2435G>A and 3488T>C in P1; 7161G>A in P2; 13886T>C in P5) and 7 depleted (i.e., 8396A>G in P1; 9247G>A and 13453C>T in P2) in LC-MBL (Fig. 5C-D, Additional file 1: Fig. S14B), consistent with a skewing of mtDNA mutations during separation from a common ancestor. To align mtDNA mutations with BCR sequences, we performed targeted sequencing of mitochondrial transcripts from single cell cDNA (*nanoranger* [25]) to obtain higher coverage and increase sensitivity of variant detection (Fig. 5E). We again observed strong concordance between monoclonal BCR sequences and enriched or depleted mtDNA mutations, which was consistent with inferred CNV changes (*del*(14q) in P1 and *tri*(12) in P5) that were exclusively identified in LC-MBL cells (Fig. 5E, G, Additional file 1: Figs. S14C-D, S15). Cases without apparent alignment of mtDNA mutations and BCR were likely explained by the stochastic loss of mtDNA mutations during cell division, a well-known aspect of mitochondrial genetics, which does not exclude a common origin. Finally, we further interrogated recurrent somatic mutations for their co-segregation with mtDNA mutations in physiologic and monoclonal B cells (Genotyping of Targeted loci with single-cell Chromatin Accessibility, GoT-ChA) [35]. In two cases (*SF3B1*^*Y632C*^ in P12 and *NOTCH1*^P2514Rfs*4^ in P24), we found a large proportion of HC-MBL cells were mutated (Fig. 5F-G, Additional file 1: Fig. S14E). This aligned with several shared but also dynamic mtDNA mutations enriched in either physiologic or monoclonal B cells (Fig. 5G, Additional file 1: Fig. S14F).

Together these results are consistent with other studies that have shown alignment of mitochondrial and somatic DNA mutations in CLL [20, 21, 36]. Further, they suggest a common origin of physiologic and monoclonal B cells, evoking observations of lymphoma-associated mutations in hematopoietic progenitors (Fig. 5H) [37, 38].

### Subclonal dynamics during MBL/CLL transition

**Fig. 6:**
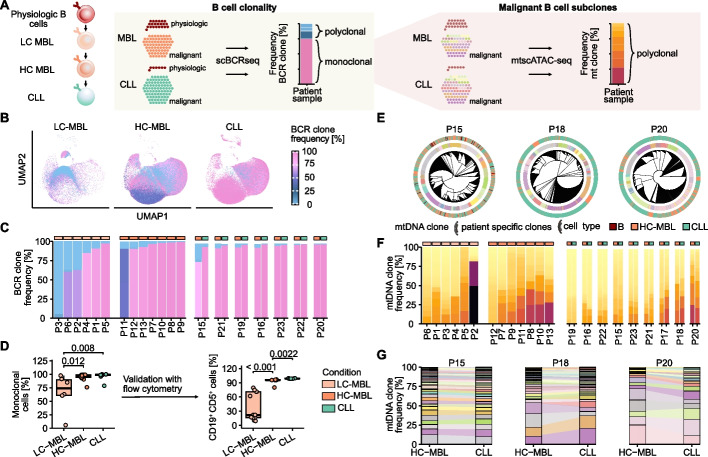
Clonal tracing in MBL/CLL transition using B cell receptor (BCR) sequences and mtDNA mutations. **A** BCR sequence-based clones are called from physiologic, premalignant (LC-/HC-MBL), and malignant B cells (CLL) using scBCR-seq to determine frequency and malignant clonal repertoire. Heteroplasmy of mtDNA mutations is calculated only for (pre-)malignant B cells. **B** Clonal expansion per LC-MBL (P1-6), HC-MBL (P7-13, P15-23), and CLL (P15-16, P19-24) based on per sample clone frequency. **C** Patient-specific BCR repertoires across LC-MBL (P1-6), HC-MBL (P7-13, P15-23), and CLL (P24). **D** Monoclonal B cell quantification in MBL/CLL from scBCR-seq data (left). Percentage of clonal CD19^+^ CD5^+^ cells by flow cytometry in an extended cohort with additional cases (*n* = 13) (right). Statistical testing using *Wilcoxon rank-sum test*. **E** Neighbor joining trees of patient-matched HC-MBL and CLL samples. The inner ring annotates mtDNA-based subclones while the other ring annotates cell types. **F** Subclonal expansion per LC-MBL (P1-6), HC-MBL (P7-13, P15-23), and CLL (P15-16, P19-24) based on per sample subclone frequency of (pre-)malignant B cells. **G** Tracking of mtDNA subclones across MBL/CLL stages for three representative cases

Having observed associations of mtDNA mutations with (pre-)malignant B cells, we asked if they could be also used to resolve subclones within monoclonal populations defined by their BCR (Fig. 6A, Additional file 1: Fig. S16A). We thus systematically evaluated BCR and mtDNA-based clonal repertoires.

First, by scBCR-seq, we found that on average 33% of physiologic, admixed B cells were polyclonal in LC-MBL (range 2–94%) (Fig. 6B-C, Additional file 1: Fig. S17A). The remarkable heterogeneity of the BCR repertoire in LC-MBL quickly contracted to a mean of 5% (range 1–24%) and 1% (0–4%) polyclonal B cells in HC-MBL and CLL. This was also associated with most physiologic B cell clones unrelated to the (pre)malignant population becoming undetectable during the MBL/CLL transition (Additional file 1: Fig. S17B). These results were consistent with analysis of flow cytometry data of 13 independent LC-MBL cases (Supplementary Methods), which showed that the number of clonal CD19^+^ CD5^+^ B cells ranged from 9 to 79% and that 5 of 13 (39%) individuals with LC-MBL already had a predominantly monoclonal B cell compartment exceeding 50% abundance (Fig. 6D, Additional file 1: Fig. S17C). Together, LC-MBL is heterogeneous regarding remaining physiologic B cells and cases with a high degree of monoclonality may relate to the increased risk of infections associated with LC-MBL [4].

Second, using mtDNA mutations, we identified on average 48 MBL/CLL subclones (Additional file 1: Fig. S17D), which ranged from 12 to 120 per case and agreed with clonal relationships discovered by neighbor joining trees (Fig. 6E). The number of subclones did not consistently differ between LC-, HC-MBL or CLL (Fig. 6F, Additional file 1: Fig. S17E). Rather, we found that some were entirely polyclonal (i.e., LC-MBL P6, HC-MBL P12 or MBL/CLL P19) and others contained large expanded subclones (LC-MBL P2, HCL-MBL P13 or MBL/CLL P20). The reasons for this observation are unclear but may reflect the stochastic genetic drift of mtDNA mutations during human aging. When comparing the frequency of individual subclones within the MBL/CLL transition, we found that 450 of 497 clones were stable (Additional file 1: Fig. S17F). In one notable exception (P20), we found 12 dynamic and 5 stable mtDNA-defined subclones (Fig. 6G). We did not observe any evidence of transcriptional or epigenetic differences between MBL/CLL matched subclones (Additional file 1: Fig. S18).

While we were unable to identify common differences in gene expression or chromatin accessibility between HC-MBL that either remained stable for > 5 years or progressed to CLL in < 5 years, the number of mtDNA-defined subclones tended to be higher in cases that underwent leukemic transition (*p* = 0.045). This suggests that the risk of MBL/CLL transition may be higher in genetically more diverse cases, a hypothesis that will require validation in larger cohorts (Additional file 1: Fig. S19A-E).

Together, we provide broad support that LC-MBL likely is a dynamic stage of CLL initiation, while the later transition from HC-MBL to overt leukemia is characterized by genetic stability in the form of persistent polyclonality with shifting, but largely stable proportions, which motivates more detailed investigations into the genetics of this early precursor state in the future.

## Discussion

CLL is the most common form of adult leukemia that has long served as a model disease for B cell lymphoma biology and has led to deep characterizations of its molecular architecture. However, the cell states in the early stages of CLL initiation are still largely unexplored, and thus it remains unclear what causes progression from MBL to CLL. Open questions include the cell-of-origin that gives rise to LC-MBL, the genetic relationships of MBL/CLL populations to physiologic B cells, but also the sequence of changes at an epigenetic and transcriptional level. Through a single-cell multi-omics approach, we have derived several novel insights into the early stages of MBL that substantially improve our understanding of CLL initiation.

First, we show that HC-MBL and CLL are molecularly almost identical. Given that the definition of the two conditions is based on a cut-off of circulating tumor cells supported by long-term survival data [39–41], this demonstrates that HC-MBL and CLL most likely do not represent biologically distinct entities. Rather, the quantification of CD19^+^ CD5^+ ^cells is a clinically important measure of disease burden that serves as a signal for the treating physician to determine the need for individual monitoring intensity. This parallels the recently proposed MDS/AML category (10–19% blasts) in the International Consensus Classification (ICC) for myeloid neoplasms, which has recognized the relevance of leukemia disease biology and that it should be interpreted together with the absolute number of circulating tumor cells [42]. By extension, this supports the notion that HC-MBL and CLL should be viewed as the same biological category along a continuum and suggests the utility of screening patients for genetic risk factors like cytogenetic abnormalities already at the time of MBL [43]. It also reinforces the need to perform large cohort studies into non-genetic mechanisms that determine whether HC-MBL remains stable or progresses, which our work was underpowered to uncover.

Second, we demonstrate the existence of CLL-like epigenetic and transcriptional core programs in LC-MBL, which is consistent with the frequent detection of copy number changes in LC-MBL. This is a remarkable observation considering the low percentage of LC-MBL that eventually become CLL and naturally raises the question what the epigenetic or transcriptional changes are that drive progression and whether they could be predictive. Our study is underpowered to address this point and does not include follow-up samples at time of CLL. However, we find heterogeneity at the level of chromatin accessibility and gene expression across the 6 studied LC-MBL cases, including higher epigenetic similarity between physiologic B cells and LC-MBL for cases with less expansion of CD19^+^ CD5^+^ cells, which suggests these differences are likely relevant and call for a deeper investigation in a larger case series. A potential explanation may be that LC-MBL in fact represents several conditions with distinct mechanisms that cannot be distinguished on flow cytometry, including age-related accumulation of expanded B cells, i.e. immunosenescence, interactions with different immune cells or infectious but also true premalignant B cell expansions.

Third, we have leveraged multiple genetic markers to interrogate the clonal relationships between physiologic B and MBL/CLL cells. The sharing of mtDNA mutations between physiologic and monoclonal B cells evokes their common ancestry [44], although we acknowledge that additional mechanisms such as independent acquisition in multiple instances, lateral mitochondrial transfer, loss of maternal variants, or early developmental events could obscure these relationships and that non-B cells were underrepresented in our data, motivating future studies with dedicated capture of these cells for genomic workup. In several cases we have additionally seen enrichment or depletion of individual mtDNA mutations in MBL/CLL compared to physiologic B cells, consistent with clonal outgrowth. These MBL/CLL-associated mtDNA mutations aligned perfectly with CNV changes or somatic mutations. This is consistent with the observation of lymphoma-initiating stem cells, for which evidence exists in CLL and follicular lymphoma (FL) [37, 45–47]. Together, it suggests a model in which a hematopoietic stem cell pool with lymphoid commitment is the founder population for MBL/CLL and provides a potential explanation for the essentially incurable nature of this disease other than through an allogeneic stem cell transplantation [48, 49]. The co-detection of mutations in *DNMT3A*, *TET2* and *ASXL1* in our cohort confirms the prevalence of clonal hematopoiesis in CLL [50] and further supports a deteriorated stem cell compartment as a driving factor of MBL. A dedicated genetic study on the origins of LC-MBL, including parallel sequencing of mitochondrial and nuclear DNA, could formally prove these hypotheses.

Our work points out opportunities for future studies. Due to the lack of routine clinical monitoring of LC-MBL and its low progression rate to CLL, complete serial collections of specimens at time of LC-MBL, HC-MBL and CLL for the same individual are lacking. Therefore, our study only provides an indirect comparison of LC- versus HC-MBL/CLL. By leveraging large prospective sample collections, it may be possible to identify such case series, which would enable direct observation of genetic, epigenetic or transcriptional changes from LC- to HC-MBL and CLL. Further, our study cohort is based on circulating mononuclear cells obtained from peripheral blood and thus, like most works, we did not have access to progenitor populations and could only indirectly infer a common ancestor of physiologic B cells and MBL/CLL. The banking of bone marrow samples of individuals with MBL or CLL would be another possibility to gain a deeper understanding of its biology, for example, by directly mapping out phylogenetic relationships with the progenitor compartment that our results suggest. As bone marrow is not required for the diagnosis of CLL, this could be investigated within the framework of a clinical study in which such material is banked.

Finally, while B cell-intrinsic factors are likely important drivers of the progression from LC-MBL to HC-MBL/CLL, by design our study was not powered to investigate immune cell function. Nevertheless, immune dysfunction is a known feature of MBL/CLL [22, 51]. It is therefore highly likely that additional extrinsic factors such as loss of immune surveillance or altered interactions with the bone marrow microenvironment including chronic BCR stimulation [52] may fuel progression to CLL. Given the increasing availability of spatial technologies [53], it will be important to investigate interactions of physiologic and monoclonal B cells within their stem cell and immune bone marrow microenvironment.

In sum, our work shows that the diagnostic distinction between HC-MBL and CLL largely does not reflect disease biology and suggests these categories to be part of an MBL/CLL continuum. Rather, LC-MBL seems to be a main inflection point during CLL pathogenesis, which motivates future investigations into its cell states and how they relate to progression to HC-MBL/CLL.

## Conclusions

Low-count monoclonal B cell lymphocytosis (MBL) already exhibits chronic lymphocytic leukemia (CLL)-like epigenetic, transcriptomic, and genetic features, with progression shaped by individual-specific cellular profiles. Mitochondrial DNA mutations further uncover genetic links between physiologic B cells, MBL, and CLL, highlighting early clonal relationships and molecular continuity in CLL initiation.

## Methods

### Patient samples

Peripheral blood samples were obtained from patients treated at Dana-Farber Cancer Institute (Boston, MA) in accordance with Institutional Review Board–approved protocols, following written informed consent. In case of white blood cell count < 25/n or absolute lymphocyte count < 25/n, B cells were enriched using the RosetteSep Human B-cell Enrichment Cocktail (Stemcell, #15064). Mononuclear cells were isolated using Ficoll-Hypaque density gradient centrifugation. After cryopreservation in 10% dimethyl sulfoxide, all samples were stored in vapor-phase liquid nitrogen until time of analysis. Classification of samples as LC-/HC-MBL or CLL were based on routine flow cytometry, white blood cell counts and presence or absence of lymphadenopathy according to guidelines of the International Workshop on CLL (iwCLL).

### Single cell RNA/BCR sequencing

Cells were resuspended in PBS containing 0.4% BSA (Invitrogen, AM2616) at a concentration of 1,000 cells/µl, and 17,000 cells were then loaded onto a Chromium Chip K (10 × Genomics, 1000286). Single-cell cDNA libraries were prepared using the Chromium Next GEM Single Cell 5′ Kit v2 (1000263) or 5’ HT Kit v2 for LC-MBL (1000356), and BCR libraries were constructed with the V(D)J Single Cell Human BCR Amplification Kit (1000253), following the manufacturer's instructions. Before sequencing, the libraries were quantified with the Bioanalyzer High Sensitivity DNA Kit (Agilent, 5067–4626), pooled, and subsequently sequenced on an Illumina NovaSeq 6000 S4 flow cell using a 28 bp read1, 90 bp read2, and 10 bp for each of the index reads.

### Single cell ATAC and ASAP sequencing

Mitochondrial single-cell ATAC-seq and ASAP-seq were carried out following established protocols [20]. Briefly, after thawing, 1–2 million cells were suspended in 50 µl of PBS supplemented with 0.04% ultrapure BSA (ThermoFisher Scientific, Cat. no. AM2618). For staining, 5 µl of Human TruStain FcX (BioLegend, Cat. no. 422302) were added, and cells were incubated for 10 min at 4 °C. Subsequently, a commercial Total-seq A 154 antibody cocktail (Additional file 2: Table S4) optimized for PBMCs (BioLegend, Cat. no. 399907) was added, and the incubation was continued for an additional 30 min at 4 °C. Fixation and permeabilization were performed according to the mtscATAC-seq method [54]. After three PBS washes containing 0.04% ultrapure BSA, cells were resuspended in 450 µl of PBS. To fix the cells, 30 µl of 16% formalin (ThermoFisher Scientific, Cat. no. 28906) was added and incubated at room temperature for 10 min. Fixation was quenched by adding 26.8 µl of 2.36 M glycine (ThermoFisher Scientific, Cat. no. 15527013) to reach a final concentration of 0.125 M in a total volume of 506.8 µl. Afterward, 700 µl of ice-cold PBS were added, followed by one more wash with PBS. Lysis was carried out by incubating the cells in 100 µl of lysis buffer for 3 min at 4 °C. Cells were then washed and finally resuspended at a concentration of approximately 4,500 cells/µl. Cell suspensions were loaded onto a Chromium Chip H (10 × Genomics, PN-1000161) with a target recovery of approximately 7,000 cells. Library construction was performed using the Chromium Next GEM Single Cell ATAC Kit v2 (1000390) in accordance with the manufacturer's protocol. To enable capture of Total-seq A oligotags, 1 µM bridge oligonucleotides were included during the barcoding step, and library preparation was carried out following the original ASAP-seq methodology [55]. After verifying library quality using the Bioanalyzer High Sensitivity DNA Kit (Agilent), pooled libraries were sequenced on an Illumina NovaSeq S2 platform, generating 50 bp paired-end reads, with an 8 bp read for index 1 and a 16 bp read for index 2.

### Lysis buffer (1 ml stock)

- 10 µl 1 M Tris–HCl (pH 7.4) (Sigma, Cat. no. T2194)
- 2 µl 5 M NaCl (Santa Cruz, Cat. no. SC-295833)
- 3 µl 1 M MgCl_2_ (ThermoFisher Scientific, Cat. no. AM9530G)
- 10 µl 10% Nonidet P40 Substitute (Sigma, Cat. no. 74385)
- 100 µl BSA 10% (Sigma, Cat. no. A3059)
- 875 µl Nuclease-free H_2_O (Promega, Cat. no. P1193)

### Washing buffer (10 ml stock)

- 100 µl 1 M Tris–HCl (pH 7.4) (Sigma, Cat. no. T2194)
- 20 µl 5 M NaCl (Santa Cruz, Cat. no. SC-295833)
- 30 µl 1 M MgCl_2_ (ThermoFisher Scientific, Cat. no. AM9530G)
- 1 ml BSA 10% (Sigma, Cat. no. A3059)
- 8.85 ml nuclease-free H_2_O (Promega, Cat. no. P1193)

### Targeted genotyping with scATAC-seq (GoT-ChA)

During library construction, locus-specific primers were spiked in as previously reported [35]. Specifically, during the GEM Generation and Barcoding reaction, 1 μL of 22.5 μM GoT-ChA primer mix was added (Additional file 2: Table S5). Amplicons were generated by nested PCR and sequenced alongside ATAC-seq libraries. Processing of the raw reads was performed using the R package Gotcha (https://github.com/landau-lab/Gotcha). A minimum of 10 reads per position and more reads supporting the mutant allele than the wildtype was considered to confidently identify the SNV of interest.

### Single cell RNA sequencing analysis

Raw reads were aligned against GRCh38 using Cell Ranger (Multiome: 7.0.0, Single Cell 5’R2-only: 7.1.0). Single cell profiles were analyzed using Seurat (4.4.0) including features being present in at least 3 cells and excluding cells of less than 200 or more than 2,500 features. To ensure high quality, only cells with mitochondrial transcript expression < 20% were retained. Data was normalized and scaled using SCTransform [56] and the principal component analysis (PCA) was calculated including all 50 principal components based on the most variable features of the data set. The neighborhood graph was constructed based on the first 10 dimensions of the PCA using Seurat's `FindCluster` function. To aid in cell type annotation, two published healthy donor PBMC datasets were utilized (H3: https://www.10xgenomics.com/datasets/human-pbmc-from-a-healthy-donor-10-k-cells-v-2-2-standard-5-0-0, H4: https://www.10xgenomics.com/datasets/10-k-human-pbm-cs-5-v-2-0-chromium-controller-2-standard-6-1-0). We used a reference-mapping approach to annotate cell types based on a healthy PBMC dataset [57] using transfer anchors as described in the ‘Multimodal reference mapping’ vignette. To verify cell types, we additionally assessed marker gene expression of M*S4A1, CD14, IL7R, CD3E, CD19, CD5, FCER2, CD79B, GNLY, NKG7, CA1* and *HBA1* [58, 59]. LC-/HC-MBL and CLL cells were annotated based on the timepoint of sampling and clonal dominance was inferred from scBCR-seq information. Single-cell copy number changes were calculated using numbat v1.4.2 using pooled T cells, monocytes, natural killer cells, dendritic cells and unannotated cells as internal reference across samples. Allele-specific information was generated using the provided `pileup_and_phase.R` script which served as an input to numbat.

### Differential gene expression analysis

Patient-specific gene expression was identified by normalizing with SCTransform [56] which accounted for our sequencing date as a covariate. Subsequently the most variable features were identified using Seurat’s `FindAllMarkers` function with a Wilcoxon rank-sum test (FDR ≤ 0.05). Genes were additionally filtered for an absolute log_2_ fold change ≥ 1 and values were aggregated as means for heatmap representation.

To identify gene expression that distinguishes physiological B cells from (pre-)malignant LC-MBL/HC-MBL/CLL cells or HC-MBL from CLL cells and to find those conserved across patients, we performed pseudo bulk analysis using DESeq2 (1.42.0) which applied a Wald test by default. The sequencing date was included as a covariate in the design formula. Genes were considered significantly differentially expressed if they showed an absolute log_2_ fold change ≥ 1 and if a -log_10_(Benjamini–Hochberg adjusted *p*-value) ≥ 10 was observed. To visualize differential gene expression at single cell resolution while correcting for batch effects, we applied scVI (0.6.8) with max_epochs = as.integer(20) for batch correction using get_normalized_expression(transform_batch = ”2022–11–18″), projecting all cells into the largest batch where all cell types were represented. Gene set enrichment analysis (GSEA) was performed using clusterProfiler (4.10.0) and the MSigDB C2 collection curated gene set.

### Analysis of single cell chromatin accessibility and protein expression profiles

Single-cell chromatin profiling data were aligned and quantified using cellranger-atac (10× Genomics, 2.0.0), employing a customized GRCh38 reference genome hard-masked for nuclear mitochondrial DNA segments (nuMTs) to enhance accuracy of mitochondrial read alignment. Downstream analyses were conducted using tailored R scripts within the ArchR (1.0.2) framework utilizing 2 LSI dimensionality reduction iterations. For cell type annotation we performed an unconstrained integration with the healthy PBMC reference data set [57]. Manual annotation via gene sores of *EBFI, PAX5, CD14, MPO, CD3D, IL7R, GNLY, NKG7, CD19, CD5, FCER2* and *CD79B*and TF motif enrichment of EBF1, PAX5, CEBPB, CEBPA, TBX21, RUNX1, and EOMES were utilized to verify the annotation. Two published healthy donor PBMC datasets were incorporated [54, 55]. LC-/HC-MBL and CLL cells cell types were annotated based on their timepoint of sampling.

Processing of TotalSeq-A libraries followed previously established methods [55]. Briefly, raw sequencing reads were reformatted from three-file to two-file FASTQ configurations compatible with kallisto (0.50.1) | bustools [60] (0.43.2) using an updated version of the ASAP-to-kite script (https://github.com/caleblareau/asap_to_kite). Feature count matrices generated via bustools were imported into R for downstream analysis using the Seurat package along with custom R scripts. Copy number changes were inferred from mtscATAC-seq profiles using a sliding-window approach as previously reported (https://github.com/caleblareau/mtscATACpaper_reproducibility) [20]. In brief, overlapping 10-Mb bins were created using a step size of 2 Mb across the whole human genome. 10× Genomics CellRanger-ATAC output files were overlapped with the bins to compute a bin by cell matrix for each LC-MBL/HC-MBL/CLL sample. Pooled LC-MBL sample T cells were used for all LC-MBL samples as a reference while pooled HC-MBL T cells CLL T cells together were used for each HC-MBL/CLL sample and bin by cell matrices were computed in an identical manner. After normalizing each cell to a consistent sequencing depth, a deviation score of the fragment number of LC-MBL/HC-MBL/CLL cells from their respective T-cell sets was calculated.

### Differential peak analysis

Pseudobulk replicates were generated per cell type and peaks were called using Macs3 (3.0.1). Subsequently, differential peaks between patients were identified using a Wilcoxon rank-sum test in ArchR’s `getMarkerFeatures()` function considering TSSEnrichment and log_10_(nFrags) as biases. Peaks were filtered for an FDR ≤ 0.1 and a log2 fold change ≥ 0.5.

Differential peak identification between physiologic B cells and LC-MBL/HC-MBL/CLL cells (Fig. 4) was performed using a Wilcoxon rank-sum test, respectively with ArchR’s getMarkerFeatures() function considering TSSEnrichment, log_10_(nFrags) and the sequencing date as biases. Peaks were interpreted significantly differential if the Benjamini–Hochberg adjusted *p*-values was ≤ 0.05 and the absolute log_2_ fold change was ≥ 1.

### Differential transcription factor motif analysis

Called peaks were annotated with transcription factor (TF) motifs using the cisbp motif set. Background peaks were identified using chromVAR (1.24.0). A motif deviation matrix was calculated using chromVAR and z-scores were extracted. A pairwise Wilcoxon rank-sum test was performed between physiologic B cells and LC-MBL/HC-MBL/CLL cells across all possible combinations. Transcription factor motifs were considered enriched/depleted with an absolute difference of z-scores > 1.5 and a Benjamini–Hochberg adjusted *p*-value < 0.05. Single-cell batch effect removal using ComBat (3.48.0) considering the sequencing date as the batch variable was utilized to generate a corrected TF motif enrichment heatmap at single-cell resolution between cell types via ComplexHeatmap [61].

### Tracking of MBL/CLL subclones using mitochondrial DNA mutations

Mitochondrial DNA mutations were extracted from aligned bam files using mgatk (0.5.6). High confidence variants were selected based on presence in > 4 cells, a variance-mean-ratio > 0.01 and strand concordance > 0.65 across longitudinal samples. Homoplasmic appearing variants across physiologic and LC-/HC-MBL and CLL cells (13886T>C, 16519T>C, 11711G>A, 2857T> C, 11435G>A, 16519T>C) hindered the identification of subclones and were excluded as well as the artifactual variant 310T>C [62].

### Identification of subclones defined by mtDNA mutations

Using Signac (1.10.0) the variants’ allele frequency (heteroplasmy) was calculated and clonotypes were called using the FindClonotypes() function consistently between patients and samples. The reliability of the identified subclones was confirmed by down sampling experiments that demonstrated the stability of subclonal definitions in 92% and 77% of cases with 75% and 50% of the data.

### Nanoranger sequencing of mitochondrial DNA mutations

Targeted enrichment of mitochondrial transcripts was performed as previously described [25, 63]. Briefly, following purification of transcripts without TSO artifacts (primers: AAO272 and bio-AAO273), a 2nd rhPCR using rhCGA_venus and a 17-primer pool (Additional file 2: Table S6) was run to amplify mitochondrial transcripts. For lowly expressed genes (*ND1*, *ND4*, *ND5*), a biotin pulldown and nested 3rd PCR were performed for further amplification. Amplicon libraries were sequenced on the Oxford Nanopore Technologies (ONT) long-read platform with the Native Barcoding Kit 24 V14 (SQK-NBD114.24) pooling 2–3 libraries per PromethION flow cell.

Single cell mtRNA reads were aligned using minimap2 (2.24-r1150-dirty) against GRCh38. Mitochondrial RNA mutations were extracted from aligned bam files using mgatk(0.7.0) in bcall mode with –nMax = 100. mtRNA mutations were then filtered based on patient-matched high-confidence mtDNA mutations discovered from mtscATAC data and as identified above. New clonotypes were called based on the mtRNA mutations’ heteroplasmy using Signac’s FindClonotype() function [64].

### Flow cytometry

The percentage of clonal B cells was calculated as the number of CD19^+^ CD5^+^ cells divided by all CD19^+^ cells to reflect dim or absent light chain expression on some MBL/CLL cases using routine flow cytometry data from the Hematology Laboratory in the Department of Pathology at Brigham and Women’s Hospital (Boston, MA). Flow cytometric immunophenotypic analysis was performed using a FACSCanto II (BD Biosciences, San Jose, CA) flow cytometer with simultaneous assessment of CD19, CD20, CD5, CD10, CD11c, CD23, CD38, CD45, kappa light chain, and lambda light chain. The flow cytometric panel used was CD19-BV421, CD20-V500, CD5-PerCp-Cy5.5, CD10-APC-R700, CD11c-BV605, CD23-APC, CD38-PE-Cy7, CD45-APC-H7, kappa-FITC, lambda-PE (all from BD Biosciences). Flow cytometric data were analyzed with FACSDiva software (BD Biosciences). Lymphocytes were first gated using CD45 vs. side scatter. Due to low expression of surface immunoglobulins in CLL, monoclonal B cells were quantified as the population of CD19^+^ CD5^+^ cells.

## Supplementary Information

Additional file 1: Fig. S1. Clinical information and mitochondrial DNA mutation characteristics. Fig. S2. Single cell ATAC-seq-based cell type annotation and IGHV mutational status. Fig. S3. Single cell RNA-seq-based cell type annotation and IGHV mutational status. Fig. S4. Chromatin tracks of CLL markers. Fig. S5. Phenotypes and frequencies of physiologic B cell subsets. Fig. S6. T cell populations across the MBL/CLL continuum. Fig. S7. Comparison of physiologic B cells and LC-MBL. Fig. S8. Identification of chromosomal aberrations in LC-MBL at single cell resolution. Fig. S9. Stable distribution of mitochondrial heteroplasmy during MBL/CLL progression. Fig. S10. Differential transcription factormotif enrichment across the MBL/CLL continuum. Fig. S11. Comparison of differential transcription factoractivity across B cells and MBL/CLL. Fig. S12. Associations of public gene expression data with CLL clinical metadata. Fig. S13. Gene set enrichments across the MBL/CLL continuum. Fig. S14. Identification and tracking of MBL/CLL subclones. Fig. S15. Mitochondrial DNA mutation heteroplasmy abundance in physiologic B and LC-MBL cells. Fig. S16. Mitochondrial DNAmutation-based clones align with BCR clonotypes. Fig. S17. Quantification of MBL/CLL subclonal dynamics. Fig. S18. Mitochondrial DNAmutation-based clone calling with mtscATAC-seq data. Fig. S19. Mitochondrial DNA mutation-based clones in stable and progressive HC-MBL.

Additional file 2: Table S1. Extended clinical information of study cohort. Table S2. Information on the 20 most frequently recurrent mitochondrial DNA mutations. Table S3. Mean heteroplasmy of 20 most frequently recurrent mitochondrial DNA mutations. Table S4. Total-seq A antibodies utilized for staining of LC-MBL cells. Table S5. Primers used for identification of somatic mutations with the GoT-ChA protocol. Table S6. Primers used for amplification of mitochondrial transcripts with nanoranger protocol.

## Data Availability

The single cell genomics data generated for this study can be access from NCBI Geo (GSE295489 [65], GSE295490 [66], GSE295491 [67]) and SRA (PRJNA1254962 [68]). The scripts used for analysis are available on GitHub [69] (https://github.com/AnjaCaRa/sc_MBL-CLL-transition_manuscript) and Zenodo [70] under an MIT license.
