# Publisher Correction: Single-cell epigenetic and transcriptomic states across the continuum of monoclonal B cell lymphocytosis to chronic lymphocytic leukemia

**DOI:** 10.1186/s13059-026-04135-6

**Published:** 2026-06-02

**Authors:** Anja C. Rathgeber, Stacey M. Fernandes, Adi Nagler, Shuqiang Li, David M. Dorfman, Lars Bullinger, Matthew S. Davids, Jennifer R. Brown, Kenneth J. Livak, Leif S. Ludwig, Catherine J. Wu, Livius Penter

**Affiliations:** 1 Berlin Institute of Health at Charité - Universitätsmedizin Berlin, Berlin, Germany; 2 Max-Delbrück-Center for Molecular Medicine in the Helmholtz Association (MDC), Berlin Institute for Medical Systems Biology (BIMSB), Berlin, Germany; 3 Department of Biology, Chemistry, Pharmacy, Freie Universität Berlin, Berlin, Germany; 4 Department of Medical Oncology, Dana-Farber Cancer Institute, Boston, MA USA; 5 Translational Immunogenomics Lab, Dana-Farber Cancer Institute, Boston, MA USA; 6 Department of Pathology, Brigham and Women’s Hospital and Harvard Medical School, Boston, MA USA; 7 Department of Hematology, Oncology, and Cancer Immunology, Berlin, Charité - Universitätsmedizin Berlin, corporate member of Freie Universität Berlin and Humboldt-Universität zu Berlin, Augustenburger Platz 1, Berlin, 13353 Germany; 8 German Cancer Consortium (DKTK), partner site Berlin, and German Cancer Research Center (DKFZ), Heidelberg, Germany; 9 National Center for Tumor Diseases (NCT), partner site Berlin, Berlin, Germany; 10 Broad Institute of Massachusetts Institute of Technology and Harvard University, Cambridge, USA; 11 Harvard Medical School, Boston, USA; 12 Berlin Institute of Health at Charité – Universitätsmedizin Berlin, BIH Biomedical Innovation Academy, BIH Charité Digital Clinician Scientist Program, Berlin, Germany

**Publisher Correction: Genome Biol 27, 175 (2026)**

**https://doi.org/10.1186/s13059-026-04072-4**

Following publication of the original article [1], a typesetting error was noticed, whereby the full “competing interest” declaration was not published. The correct declaration is:

C.J.W. is an equity holder of BioNTech, Inc, receives research funding from Pharmacyclics, and is a SAB member of Repertoire, Nature’s Toolbox, Aethon Therapeutics, and Adventris.

K.J.L. reports equity in Standard BioTools Inc. and serves on the scientific advisory board for MBQ Pharma Inc.

L.B. received honoraria from AbbVie, Amgen, Astellas, Bristol Myers Squibb, Celgene, Daiichi Sankyo, Gilead, Hexal, Janssen, Jazz Pharmaceuticals, Menarini, Novartis, Otsuka, Pfizer, Roche, and Sanofi; research funding from Bayer, Jazz Pharmaceuticals.

M.S.D. has received institutional research funding from AstraZeneca and Ascentage Pharma,; and received personal consulting income from AbbVie, Ascentage Pharma, AstraZeneca, BeOne Medicines, Bristol Myers Squibb, Eli Lilly, Genentech, Genmab, Janssen, Merck, Nuvalent,and Schrӧdinger, and receives royalties from UpToDate.

J.R.B. has served as a consultant for Abbvie, Acerta/Astra-Zeneca, Alloplex Biotherapeutics, BeiGene, Bristol-Myers Squibb, EcoR1, Galapagos NV, Genentech/Roche, Grifols Worldwide Operations, InnoCare Pharma Inc, Kite Pharma, Loxo/Lilly, Magnet Biomedicine, Merck, Numab Therapeutics, Nurix Therapeutics, Pfizer, Pharmacyclics; received research funding from BeiGene, Gilead, iOnctura, Loxo/Lilly, MEI Pharma, Nagoon Therapeutics, and TG Therapeutics; serves on the Data Safety Monitoring Board for Grifols Therapeutics; and receives royalties from UpToDate.

The Broad Institute has filed for patents relating to the use of technologies described in this paper, where L.S.L. is named an inventor (US patent applications 17/251,451 and 17/928,696).

L.S.L is a Guest Editor for Genome Biology’s “Leveraging mitochondria to understand health and disease” collection but was not involved in the editorial process of this manuscript.

All other authors do not have any relevant conflict of interest.

The original article [1] has been updated.
