# Supplementary material for: Single-cell epigenetic and transcriptomic states across the continuum of monoclonal B cell lymphocytosis to chronic lymphocytic leukemia
Source: Genome Biol. 2026 Apr 15;27:175. doi: 10.1186/s13059-026-04072-4 (PMC13192028; doi:10.1186/s13059-026-04072-4)
Supplement: Supplementary file 1 — Additional file 1: Fig. S1. Clinical information and mitochondrial DNA mutation characteristics. Fig. S2. Single cell ATAC-seq-based cell type annotation and IGHV mutational status. Fig. S3. Single cell RNA-seq-based cell type annotation and IGHV mutational status. Fig. S4. Chromatin tracks of CLL markers. Fig. S5. Phenotypes and frequencies of physiologic B cell subsets. Fig. S6. T cell populations across the MBL/CLL continuum. Fig. S7. Comparison of physiologic B cells and LC-MBL. Fig. S8. Identification of chromosomal aberrations in LC-MBL at single cell resolution. Fig. S9. Stable distribution of mitochondrial heteroplasmy during MBL/CLL progression. Fig. S10. Differential transcription factormotif enrichment across the MBL/CLL continuum. Fig. S11. Comparison of differential transcription factoractivity across B cells and MBL/CLL. Fig. S12. Associations of public gene expression data with CLL clinical metadata. Fig. S13. Gene set enrichments across the MBL/CLL continuum. Fig. S14. Identification and tracking of MBL/CLL subclones. Fig. S15. Mitochondrial DNA mutation heteroplasmy abundance in physiologic B and LC-MBL cells. Fig. S16. Mitochondrial DNAmutation-based clones align with BCR clonotypes. Fig. S17. Quantification of MBL/CLL subclonal dynamics. Fig. S18. Mitochondrial DNAmutation-based clone calling with mtscATAC-seq data. Fig. S19. Mitochondrial DNA mutation-based clones in stable and progressive HC-MBL. [file 13059_2026_4072_MOESM1_ESM.pdf]

Supplementary Data

Single-cell epigenetic and transcriptomic states across the continuum of monoclonal B cell lymphocytosis to chronic lymphocytic leukemia

<u>Inventory:</u>

- Supplementary Methods
- Supplementary Figures 1-19

Supplementary Methods

Single Cell Multiome (ATAC-seq, gene expression and surface protein expression)

Used for samples from MBL/CLL P17 and P18. Cells were stained with Total-seq A antibodies, fixed, and permeabilized as described below, and loaded onto a Chromium chip J (10x Genomics, 1000234). Single cell RNAseq libraries and ATACseq libraries were prepared using Chromium Next GEM Single Cell Multiome ATAC + Gene Expression Reagent Bundle (10x Genomics,1000283), the surface protein expression libraries were made following the original ASAP-seq methodology.[1] The RNAseq and CITEseq libraries were sequenced using Illumina NovaSeq 6000 system S4 flow cell, and the sequencing parameters were 28 bp read 1, 90 bp read 2, and 10 bp for each of the index reads. The ATACseq libraries were sequenced using Illumina NovaSeq 6000 system S2 flow cell, and the sequencing parameters were 50 bp read 1, 49 bp read 2, and 8 bp for index 1 and 24 bp for index 2.

B cell receptor sequencing analysis

BCR repertoire analysis and identification of CLL: CDR3 sequences were called with enclone embedded into cellranger (7.1.0) using vdj_GRCh38_alts_ensembl-5.0.0. Clonotypes characterized by multiple different IGH, IGK or IGL chains were filtered out and considered doublets. Clonotypes were matched in longitudinal samples based on CDR3 sequence. To account for clonotypes from incomplete IG chain set detection, a custom python script was created to aggregate clonotypes. Clonotypes were merged when their CDR3s matched and their detected IG chain sets represented strict subsets of more complete clonotypes with identical CDR3s. The most abundant clonotypes were considered MBL/CLL, while other less frequent clonotypes were annotated as residual physiological B cells.

IGHV mutation status calling

Sanger sequencing-based clinical *IGHV* mutation status data have been available only for a subset of patient samples in this cohort. To stratify our cohort also for the other samples we bioinformatically assessed the *IGH* gene using the dominant BCR clone sequence information from scBCR-seq which we obtained from cellranger outputs. The dominant clone per sample was identified from the clonotypes.csv file, while its matched IGH sequence (fwr1_nt, cdr1_nt, fwr2_nt, cdr2_nt, fwr3_nt, cdr3_nt, fwr4_nt) was extracted from the consesuns_annotations.csv file. To assess the IGHV mutational status, we used a human germline reference (IMGT human V genes (F+ORF+in-frame P), IMGT human D genes (F+ORF), IMGT human J genes (F+ORF+in-frame P)) to compare to with Ig BLAST (https://www.ncbi.nlm.nih.gov/igblast/) as advised by official ERIC guidelines[2]. Ig BLAST parameters were set as follows: Program: blastn, V gene mismatch penalty: -1, Min D gene nucleotide matches: 5, D gene mismatch penalty: -2, Min required V gene length: 9, Min required J gene length: 0, J gene mismatch penalty: -2. Deviations of ≥ 2% from the germline reference were considered to be mutated, while < 2% were considered to be unmutated[3]. This approach for obtaining *IGHV* mutational status was validated by the clinical Sanger sequencing data from the study cohort where available.

TF activity calculation

Transcription factor activity was inferred from scRNA-seq using the decoupleR package with the CollecTRI dataset from the OmniPath database. This enabled us to link changes in TF motifs from scATAC-seq data to gene expression changes. We used the weighted mean (WMEAN) approach with 90 iterations and pseudo bulked TF activities per patient and cell type. Changes in transcription factor activity were assessed for all TFs which were previously found to have differential TF motif enrichment in our comparisons of LC-MBL/HC-MBL and CLL cells to residual physiologic B cells.

Supplementary Figures

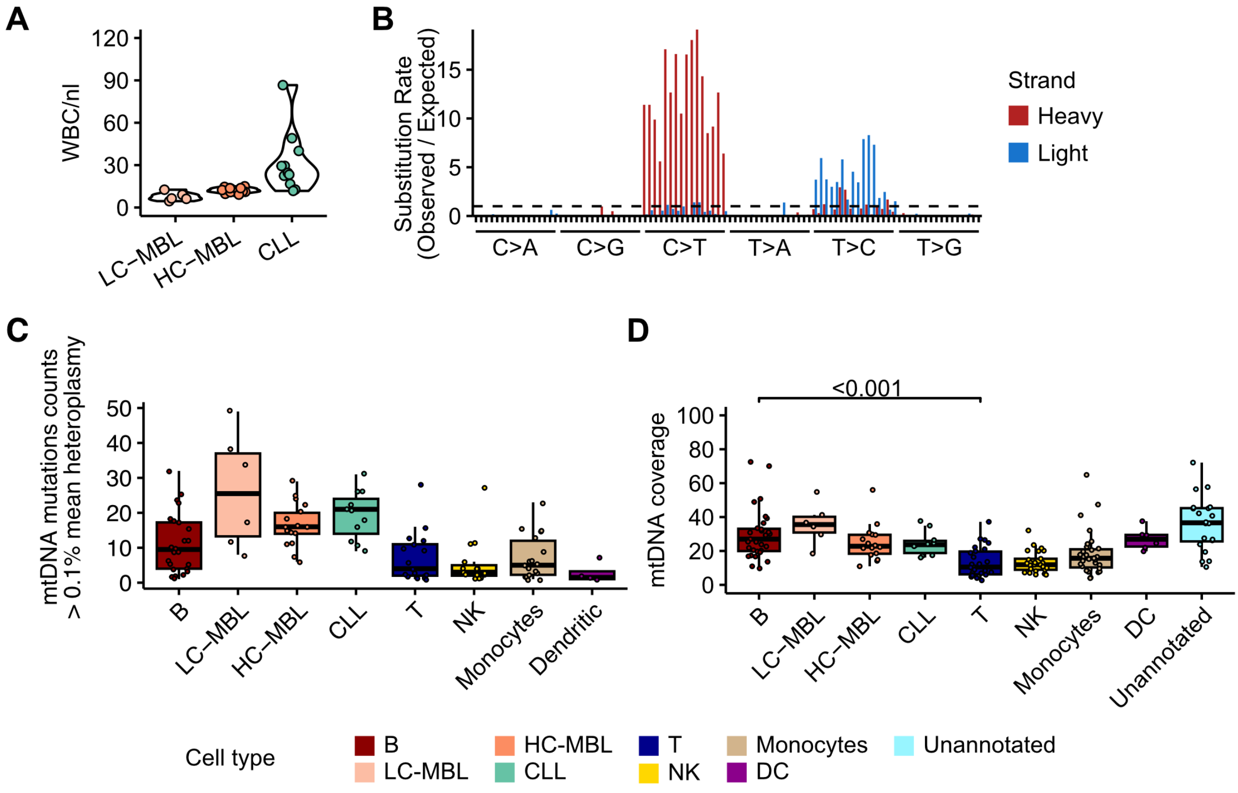

Fig. S1 Clinical information and mitochondrial DNA (mtDNA) mutation characteristics.
A White blood cell counts across all samples per condition.
B Mutational signatures of all 677 mtDNA mutations detected across all 34 samples and cell types.
C Mean mtDNA mutation counts with >0.1 % mean heteroplasmy across cell types per patient.
D Mean mtDNA coverage across cells per sample and cell type.

Statistical testing using *Wilcoxon rank sum test.*

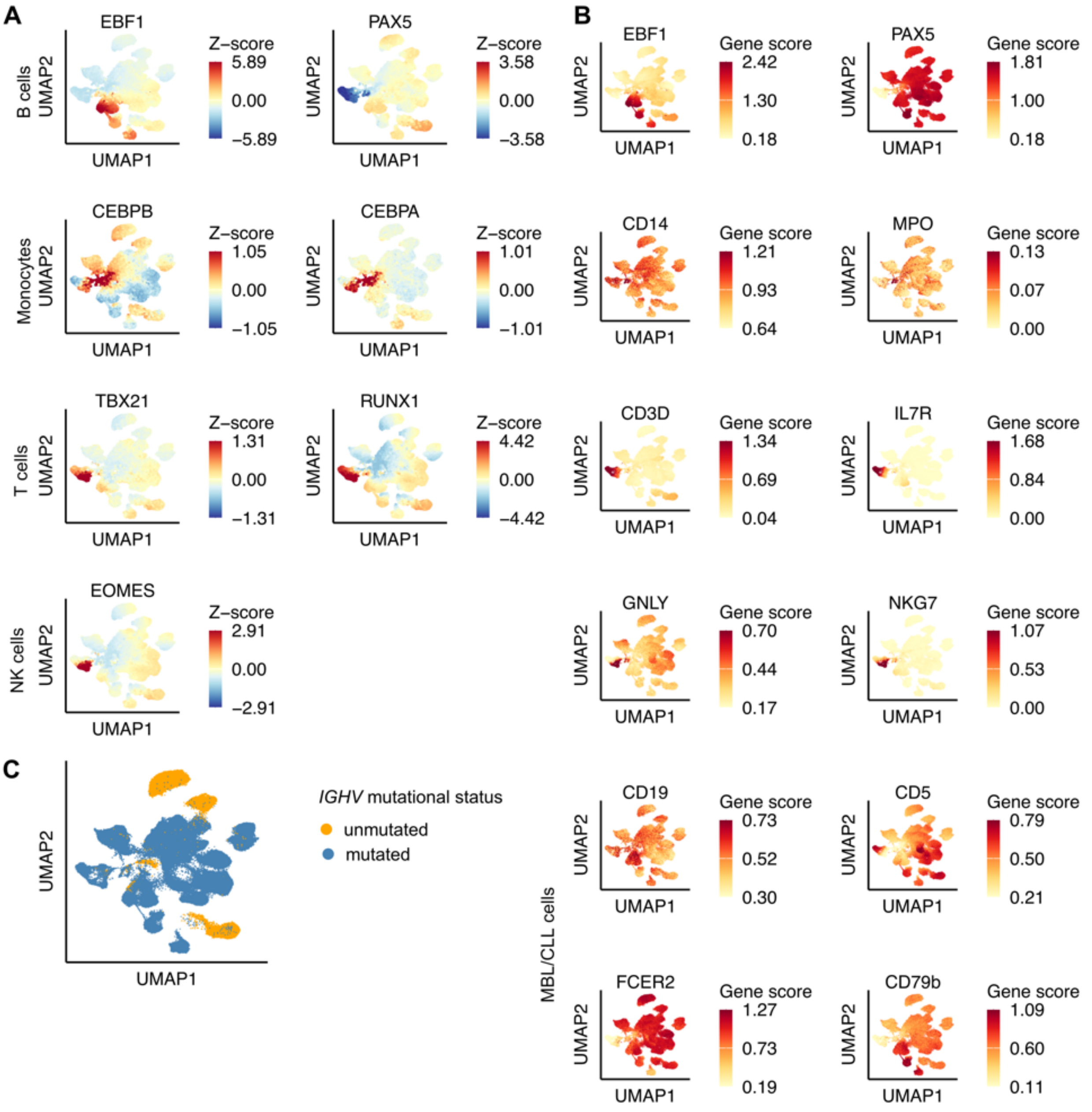

Fig. S2 Single cell ATAC-seq (scATAC-seq)-based cell type annotation and IGHV mutational status.
A UMAPs of Transcription Factor (TF) motif enrichment for B cells, monocytes, T cells and NK cells.
B UMAPs of gene scores (imputed gene expression) of cell type marker genes identifying B cells, monocytes, T cells, NK cells and MBL/CLL cells (top to bottom).
C UMAP of *IGHV* mutational status of all LC-/HC-MBL and CLL samples, where information was available.

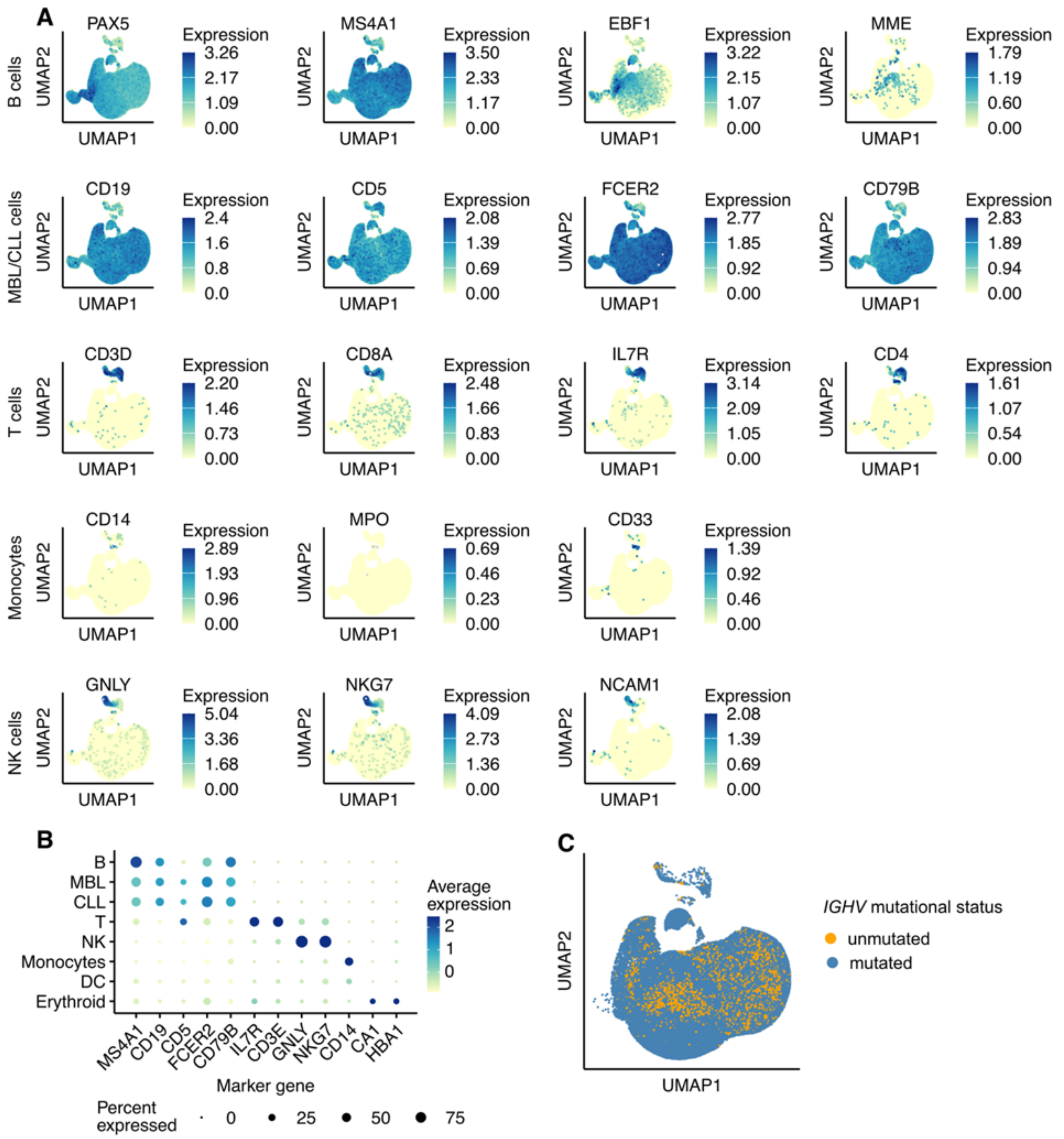

Fig. S3 Single cell RNA-seq (scRNA-seq)-based cell type annotation and *IGHV* mutational status.
A UMAPs of marker gene expression B cells, MBL/CLL cells, T cells, monocytes, and NK cells.
B Mean expression of marker gene in cell type annotation used.
C UMAP of *IGHV* mutational status of all LC-/HC-MBL and CLL samples, where information was available.

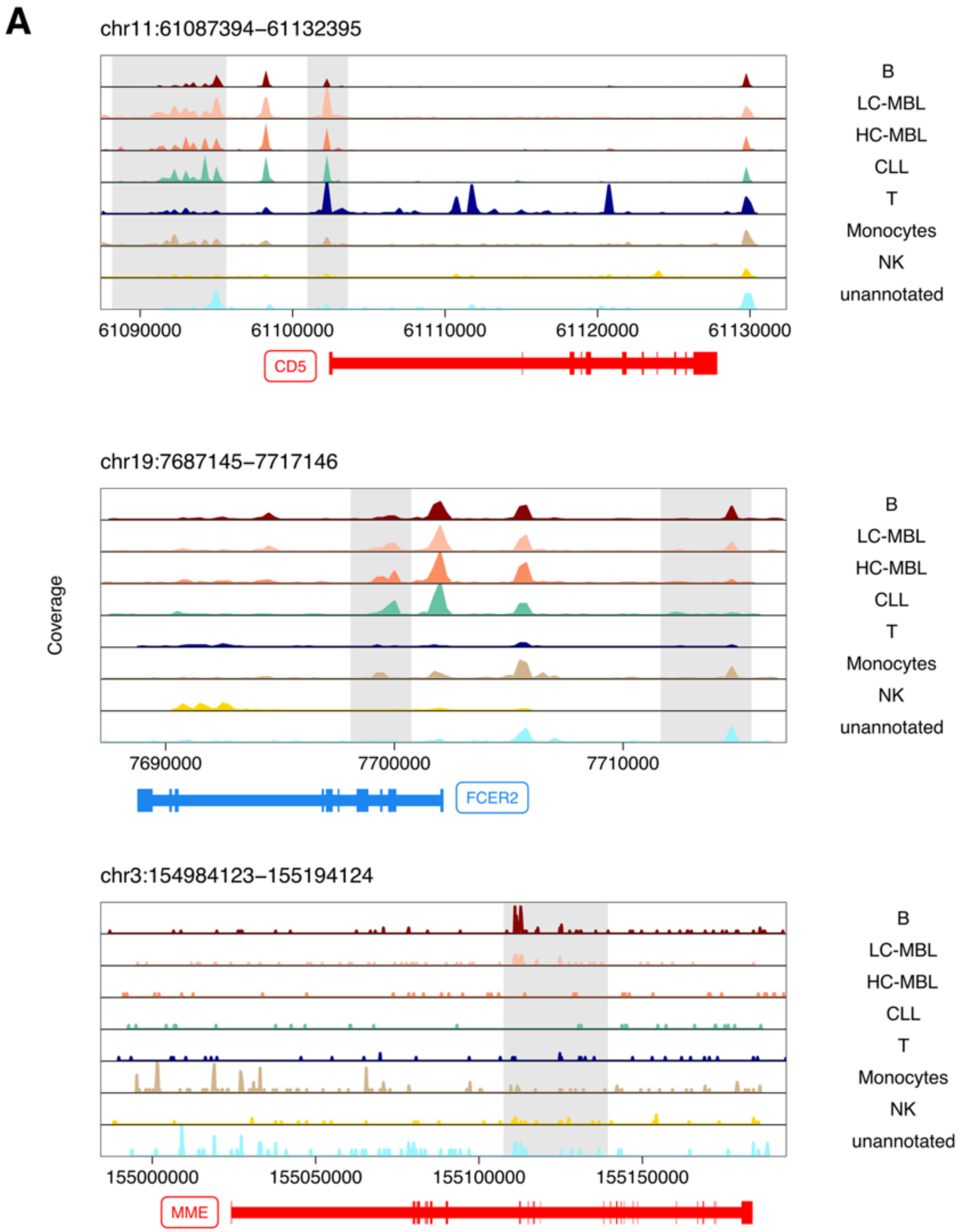

Fig. S4 Chromatin tracks of CLL markers.
A Cell type specific chromatin accessibility for the canonical CLL marker genes *CD5*, *FCER2* (encoding CD23), and *MME* (CD10), from top to bottom.

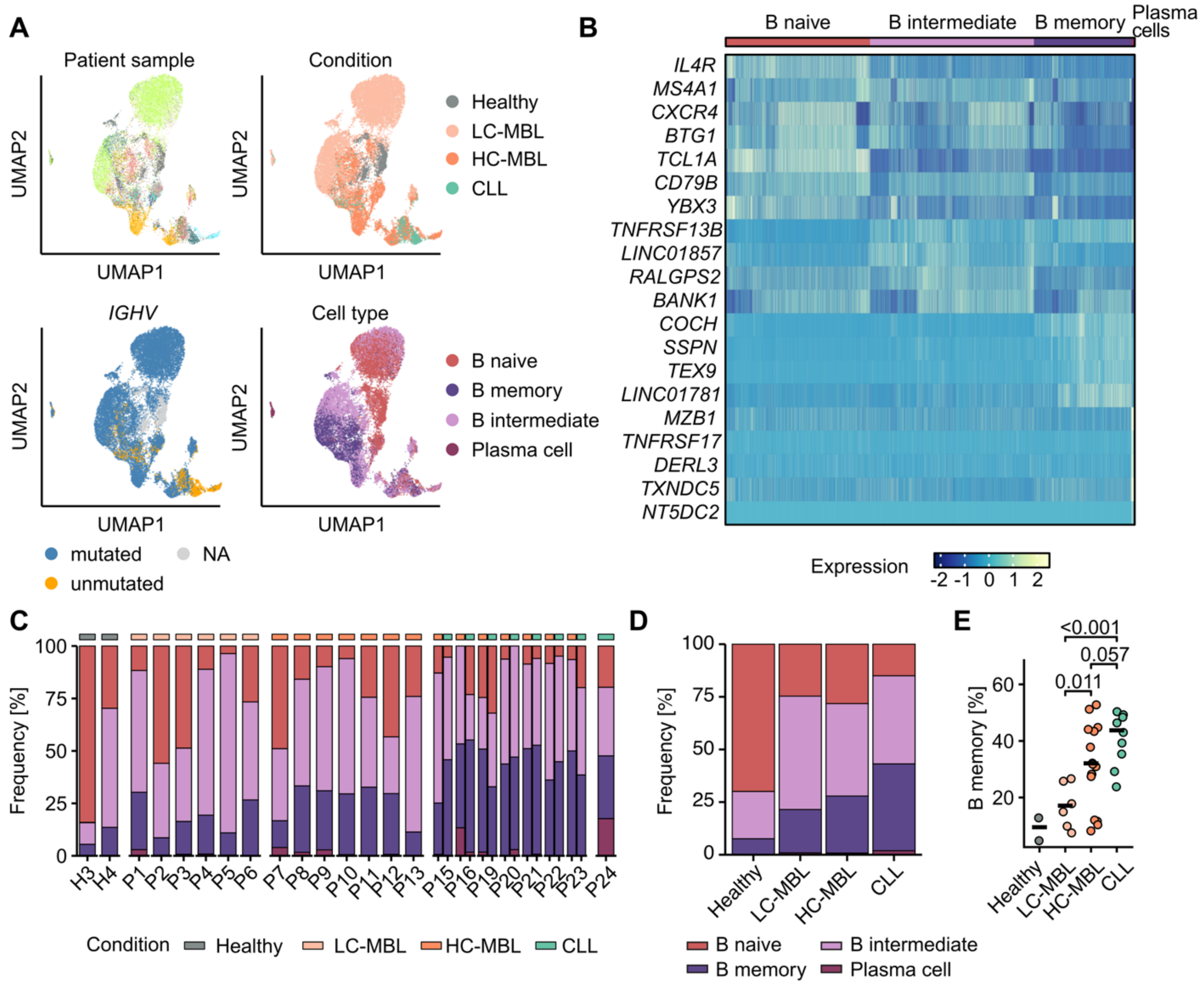

Fig. S5 Phenotypes and frequencies of physiologic B cell subsets.

A UMAP representations of physiologic B cells gene expression profiles from scRNA-seq annotated by label transfer. Representation by patient sample (colors correspond to main Figure 2), sample condition, *IGHV* mutational status and B cell subtype annotation.

B Cell type marker expression.

C Cell type proportion across healthy controls, LC-MBL, HC-MBL and CLL.

D Median cell type composition per condition.

E Percentage of memory B cells across healthy controls, LC-MBL, HC-MBL and CLL.

Statistical testing with Student t-test.

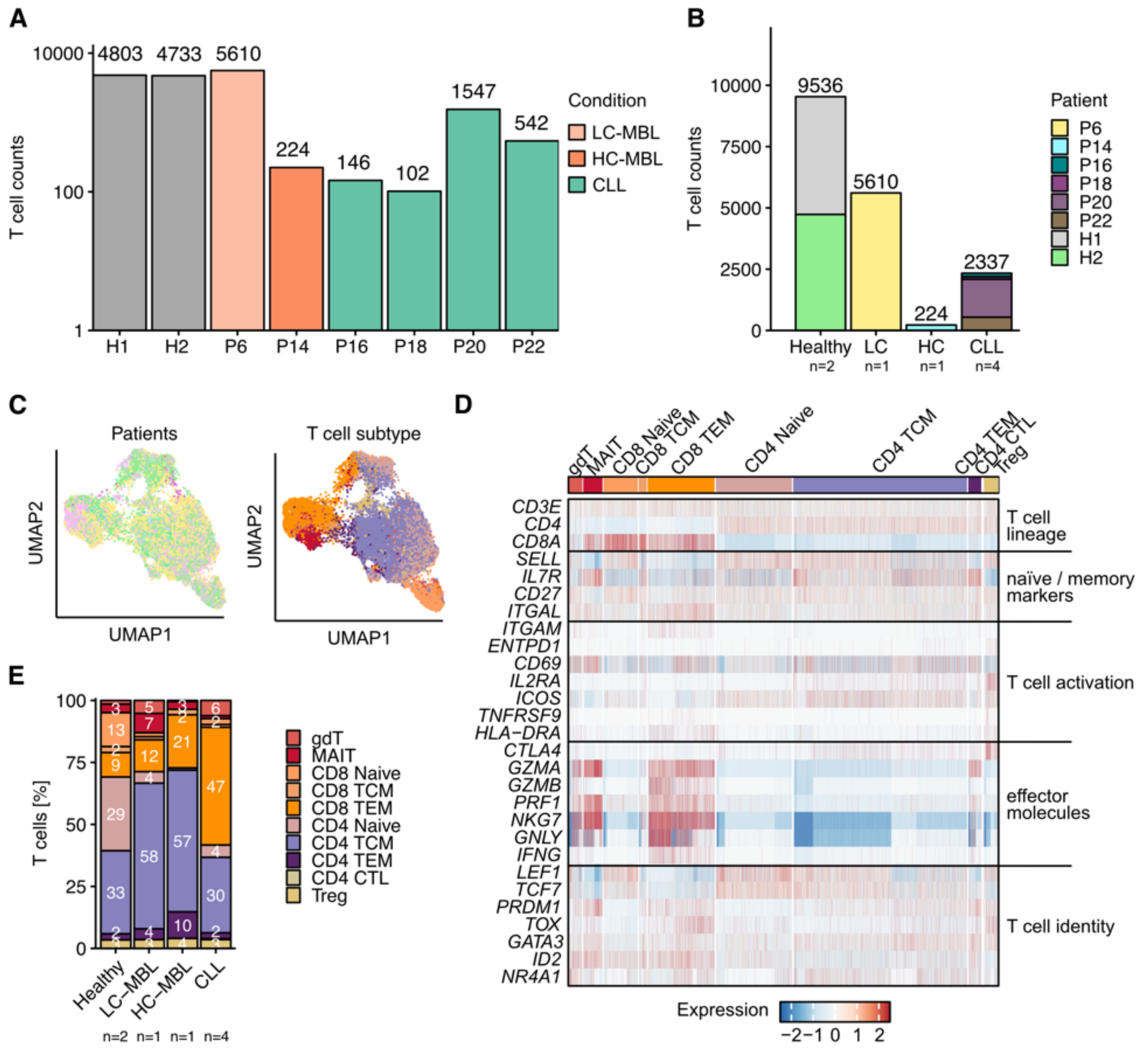

Fig. S6 T cell populations across the MBL/CLL continuum.
A Total T cell counts per healthy gene expression control and patient sample.
B Total T cell counts per condition.
C UMAP of T cells colored by samples from A and B (left). UMAP of all T cells annotated by cell type from label transfer of healthy PBMC reference data set (right).
D Cell type marker expression across T cell subsets.
E Quantification of T cell subsets from panel D per condition.
CTL - cytotoxic T lymphocyte, gdT - γ-δ T cells, MAIT - mucosa-associated invariant T cells, TCM - central memory T cell, TEM - effector memory T cell, Treg - regulatory T cells

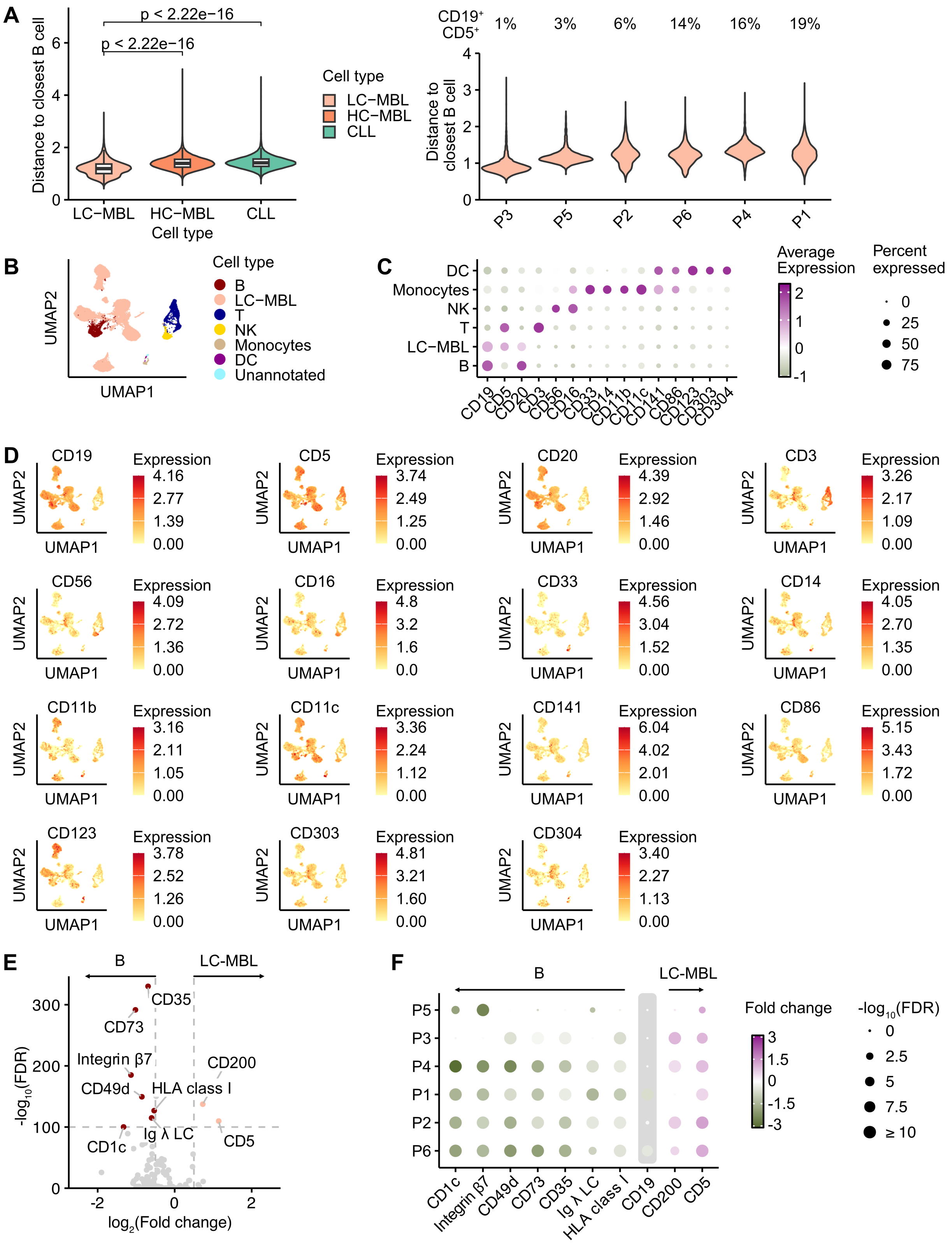
A
p < 2.22e−16
p < 2.22e−16
Distance to closest B cell
LC−MBL
HC−MBL
CLL
Cell type
Cell type
LC−MBL
HC−MBL
CLL
CD19+ CD5+
1%
3%
6%
14%
16%
19%
Distance to closest B cell
P3
P5
P2
P6
P4
P1
B
Cell type
B
LC−MBL
T
NK
Monocytes
DC
Unannotated
UMAP1
UMAP2
C
DC
Monocytes
NK
T
LC−MBL
B
CD19
CD5
CD20
CD3
CD56
CD16
CD33
CD14
CD11b
CD11c
CD141
CD86
CD123
CD303
CD304
Average Expression
Percent expressed
D
CD19
CD5
CD20
CD3
CD56
CD16
CD33
CD14
CD11b
CD11c
CD141
CD86
CD123
CD303
CD304
Expression
E
B
LC-MBL
CD35
CD73
Integrin β7
CD200
CD49d
HLA class I
Ig λ LC
CD1c
CD5
-log10(FDR)
log2(Fold change)
F
B
LC-MBL
P5
P3
P4
P1
P2
P6
CD1c
Integrin β7
CD49d
CD73
CD35
Ig λ LC
HLA class I
CD19
CD200
CD5
Fold change
-log10(FDR)

Fig. S7 Comparison of physiologic B cells and LC-MBL.

A Euclidean distance from LC-MBL/HC-MBL and CLL cells to the closest residual physiologic B cell (left). Patient-resolved distances ordered by increasing % $CD19^{+}$ $CD5^{+}$ B cells (right).

Statistical testing using *Wilcoxon rank sum test*.

B Weighted-nearest-neighbors (WNN) UMAP of combined scATAC-seq and CITE-seq data with projection of cell type annotation from scATAC-seq.

C Cell type specific protein marker expression as identified from Total-seq A antibody cocktail.

D WNN-UMAPs with projection of protein marker expression for B, LC-MBL cells, T cells, NK cells, monocytes and dendritic cells.

E, F Identification of differential phenotypic surface marker expression between healthy B cells of LC-MBL patients and respective LC-MBL cell population (E). Wilcoxon rank sum test with Benjamini-Hochberg correction. Patient-resolved surface marker expression of significantly up-/down-regulated proteins (F).

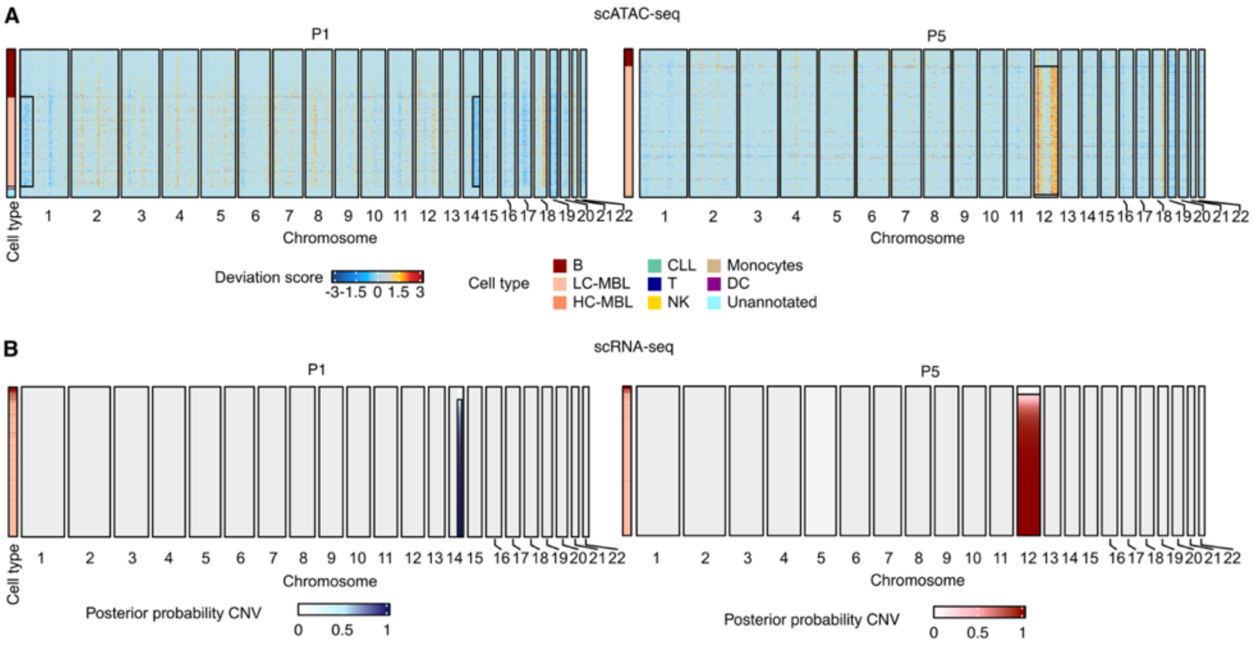

Fig. S8 Identification of chromosomal aberrations in LC-MBL at single cell resolution.

A Copy number changes calculated from scATAC-seq data for each cell type for P1 (left) show deletions in chromosome 1 and a *del*(14q) as well as a *tri*(12) in P5 (right) highlighted. LC-MBL T cells were used as a reference offset.

B Copy number changes calculated from scRNA-seq data in LC-MBL and physiologic B cells in P1, confirming *del*(14q) and *tri*(12) in P5 identified from scATAC-seq data. T cells, monocytes, NK cells, DCs, and unannotated cells were used as a reference offset.

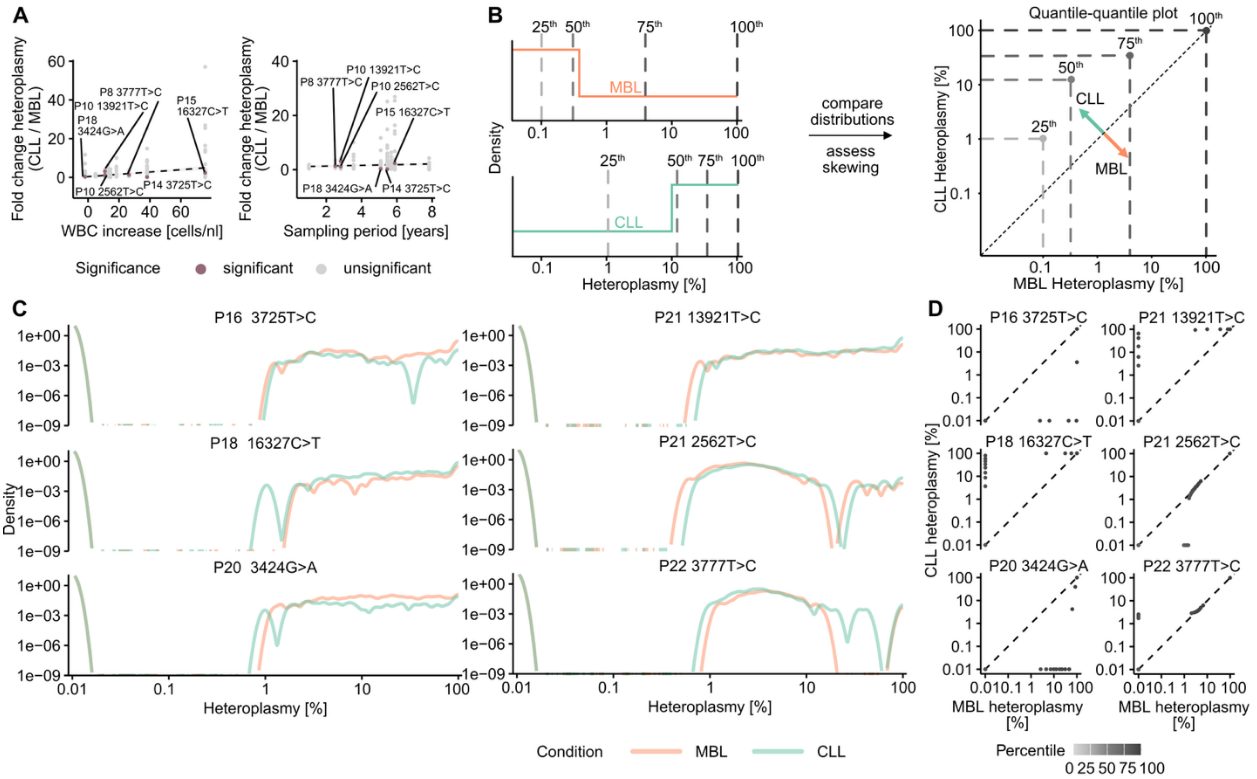

Fig. S9 Stable distribution of mitochondrial heteroplasmy during MBL/CLL progression.

A Mean heteroplasmy changes compared to white blood cell count (WBC) increase between HC-MBL and CLL stage (left) and time between HC-MBL and CLL sample (right). Statistical significance was assessed using a Kolmogorov–Smirnov test of mtDNA heteroplasmy distributions for mutations detected in ≥ 100 cells at either the MBL or CLL stage, with Benjamini-Hochberg correction (FDR ≤ 0.05). Linear regressions are depicted as dashed lines with a 95% confidence interval in light grey.

B Schematic explaining a two-sample Q-Q plot for the assessment of heteroplasmy distributions. Distributions of the two samples being compared (left), here MBL and CLL, are summarized using equal quartiles of heteroplasmy. Corresponding quartiles of both distributions are then plotted against each other in a scatterplot allowing changes in distributions to be observed at the desired quantile resolution. While shifts towards the y-axis indicate a skewed heteroplasmy in corresponding quantiles in the CLL stage and shifts towards the x-axis indicate a skewing of heteroplasmy in the corresponding quantiles at the MBL stage. Equal distribution quantiles would align on the diagonal.

C All statistically significantly different heteroplasmy distributions between patient-matched HC-MBL and CLL samples as identified via the Kolmogorov–Smirnov test of mtDNA heteroplasmy distributions for mutations detected in ≥ 100 cells at either the MBL or CLL stage, with Benjamini-Hochberg correction (FDR ≤ 0.05). **D** Q-Q plots for all statistically significantly different heteroplasmy distributions between patient-matched HC-MBL and CLL samples.

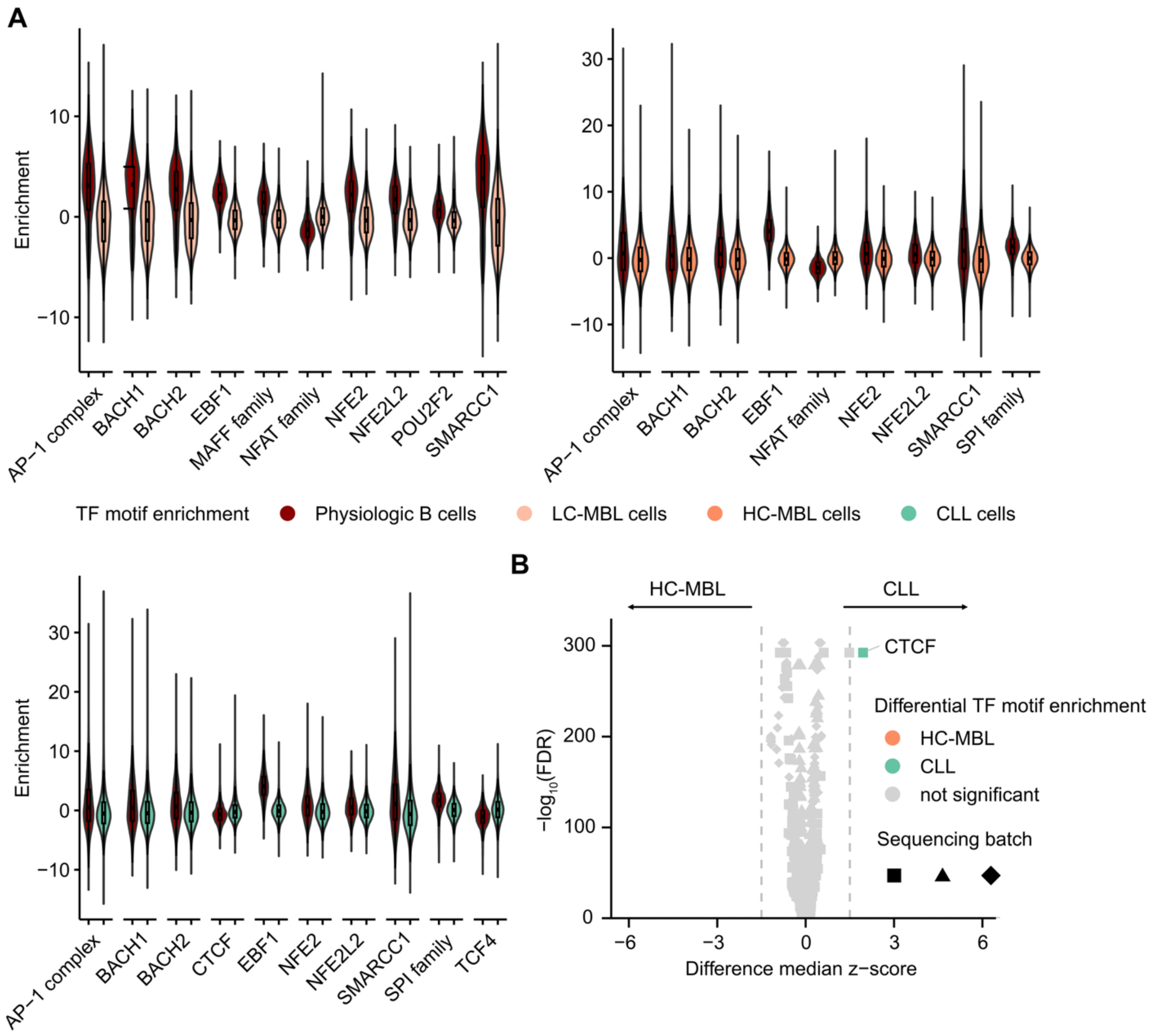

Fig. S10 Differential transcription factor (TF) motif enrichment across the MBL/CLL continuum.
A Mean significant TF motif enrichment across samples and TF families from pairwise comparisons of physiologic B cells (from LC-MBL samples) vs. LC-MBL cells (top left), physiologic B cells (from HC-MBL and CLL samples) vs. HC-MBL cells (top right) and physiologic B cells (from HC-MBL and CLL samples) vs. CLL cells (bottom right). **B** Pseudo bulk TF motif enrichments between HC-MBL and CLL samples. Significance was assessed using a Wilcoxon rank sum test (Benjamini-Hochberg correction FDR ≤ 0.5, thresholds as dashed lines with an absolute difference of median z-scores > 1.5)

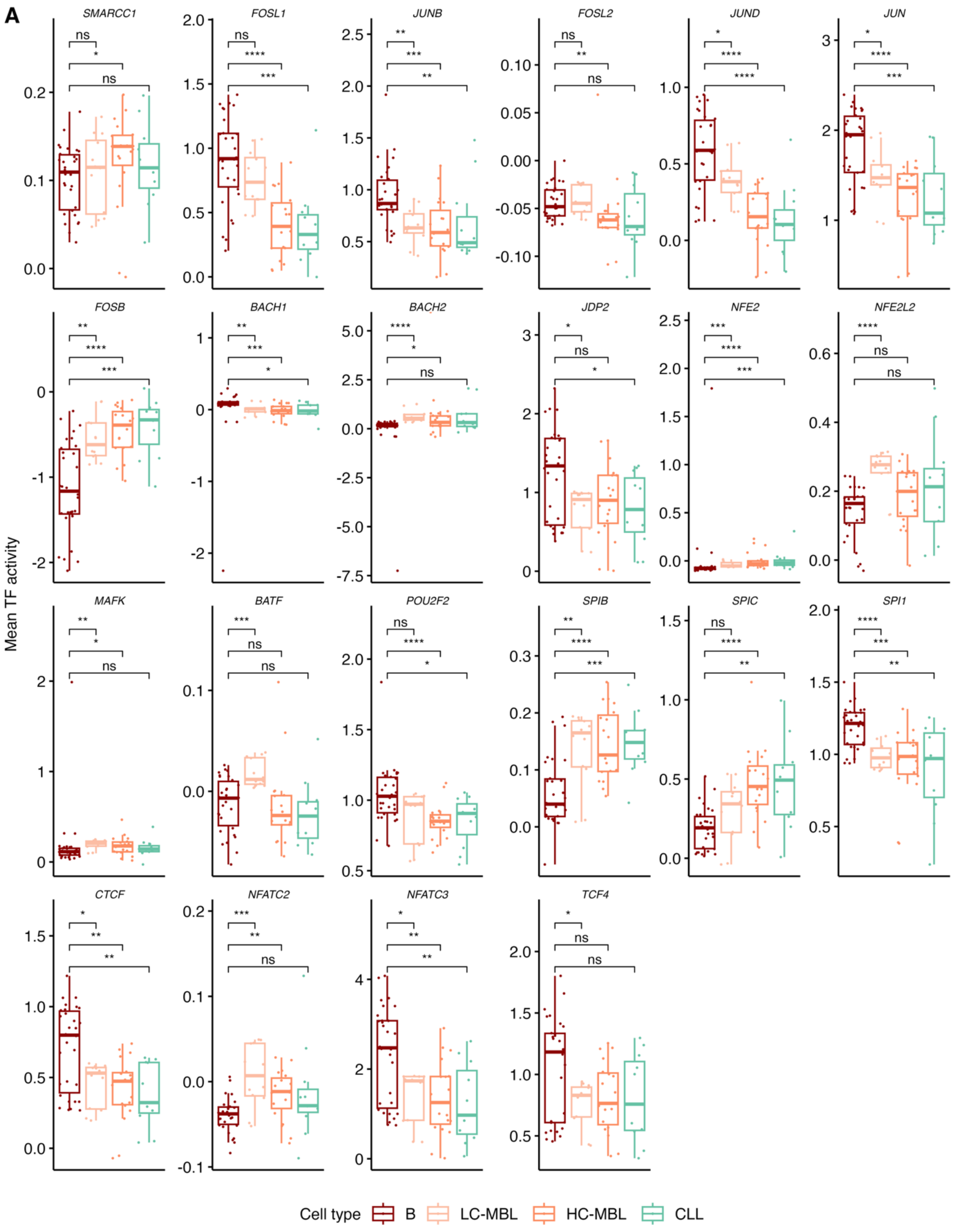

Fig. S11 Comparison of differential transcription factor (TF) activity across B cells and MBL/CLL.
The mean TF activity was inferred from single cell RNA-seq data. TFs from *SMARCC1* to *POU2F2* (row wise) had reduced motif enrichment in scATAC-seq profiles in MBL/CLL compared to residual physiologic B cells. TFs from *SPIB* to *TCF4* were enriched in MBL/CLL on scATAC-seq. Statistical testing using *Wilxocon rank sum test.*

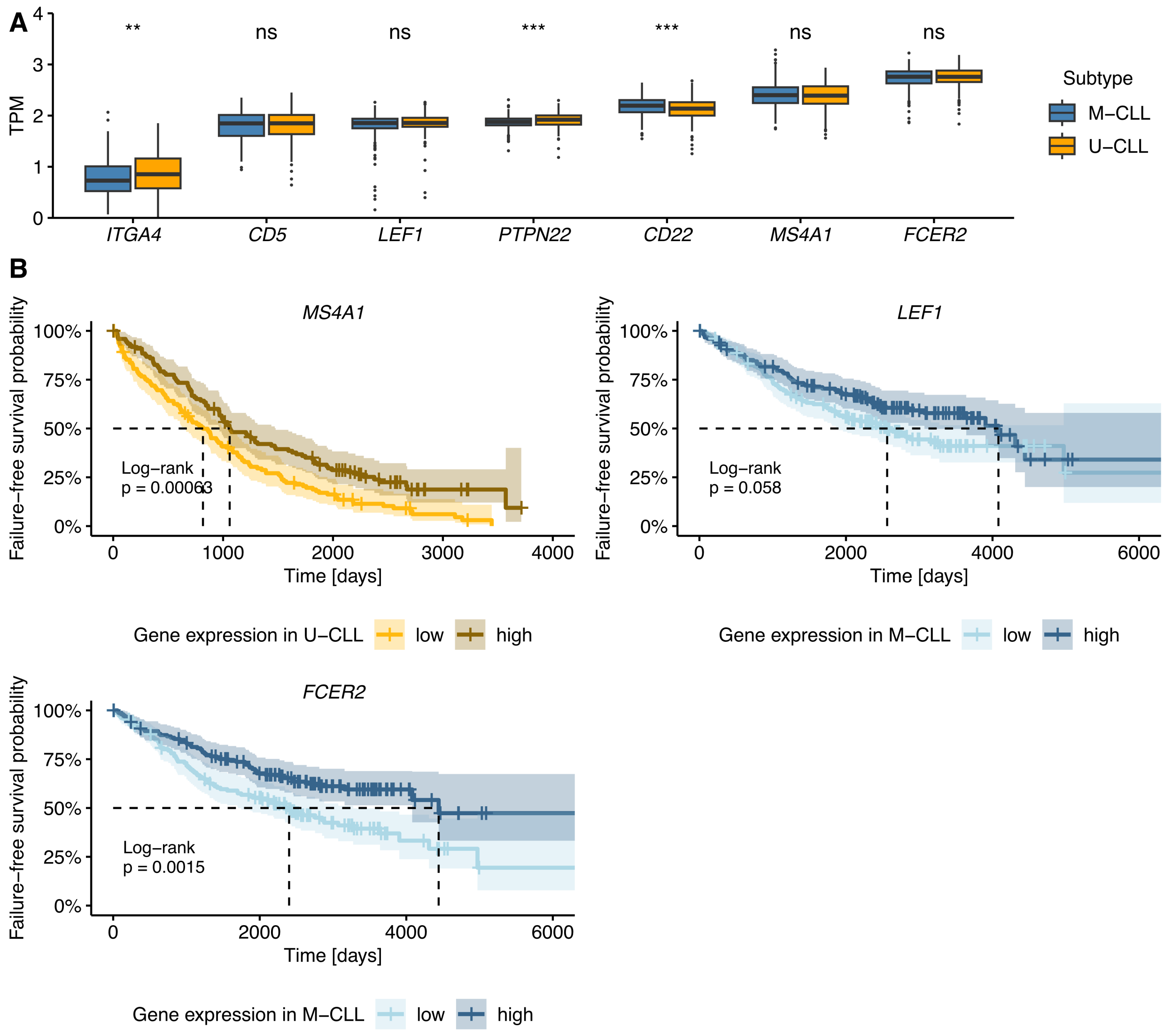

Fig. S12 Associations of public gene expression data with CLL clinical metadata.
Differentially expressed genes identified as part of the CLL-associated core program were investigated for associations with clinical outcomes reported in *Knisbacher et al., Nature Genetics 2022.*
A Differential gene expression in IGHV mutated (M-CLL) and IGHV unmutated CLL (U-CLL).
B Failure-free survival probability in cases with higher than median (high) or lower than median (low) expression of the indicated genes. Statistical testing using *log-rank test.*

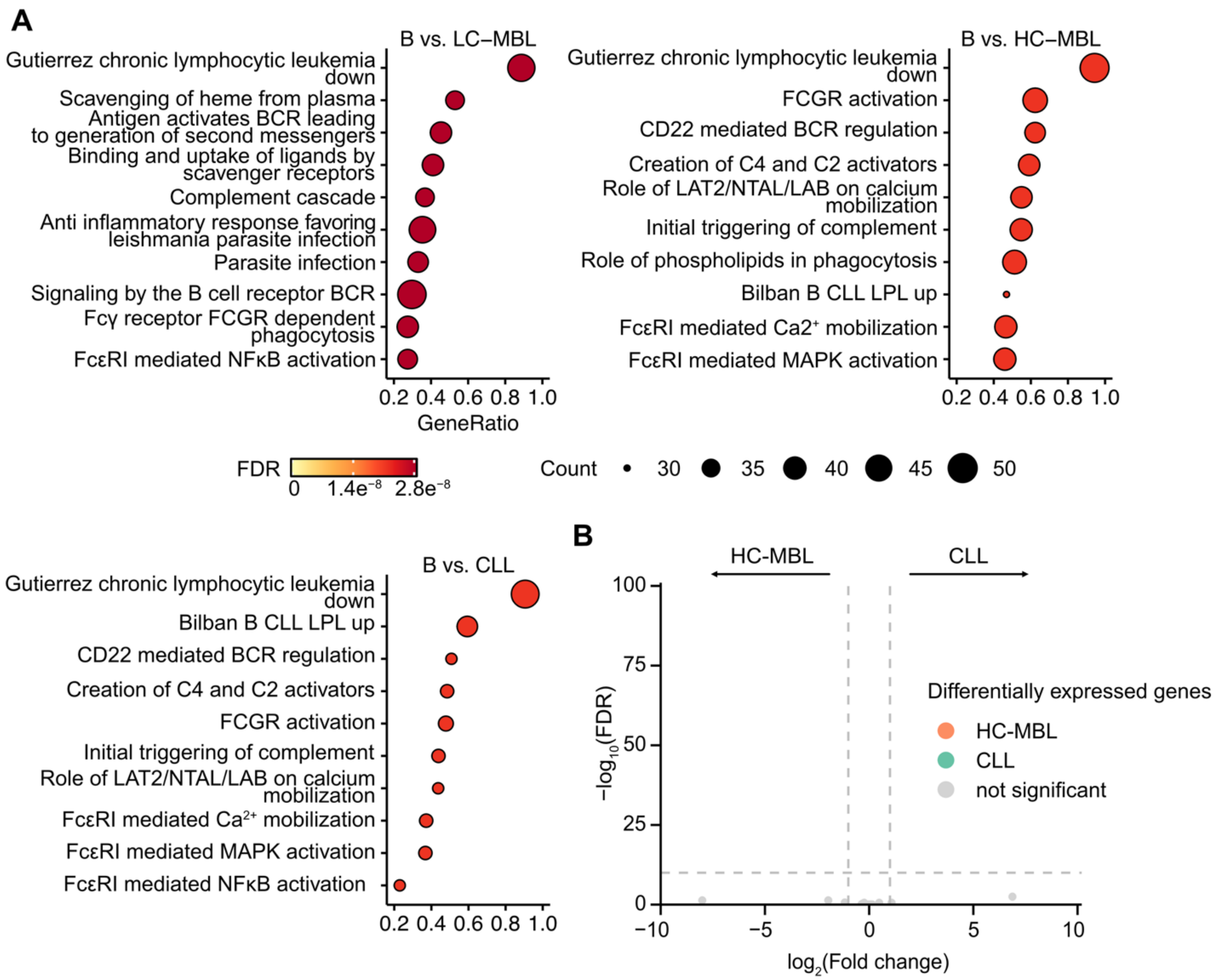

Fig. S13 Gene set enrichments across the MBL/CLL continuum.

A Gene set enrichment analysis **(**GSEA) of MSigDB C2 collection on pairwise comparisons of physiologic B cells (from LC-MBL samples) vs. LC-MBL cells (top left), physiologic B cells (from HC-MBL and CLL samples) vs. HC-MBL cells (top right) and physiologic B cells (from HC-MBL and CLL samples) vs. CLL cells (bottom left).

B Pseudo bulk gene expression between HC-MBL and CLL samples. Significance was assessed using a Wald test, and thresholds are depicted as dashed lines with an absolute average log2 fold change > 1 and -log10(FDR) cut off > 10.

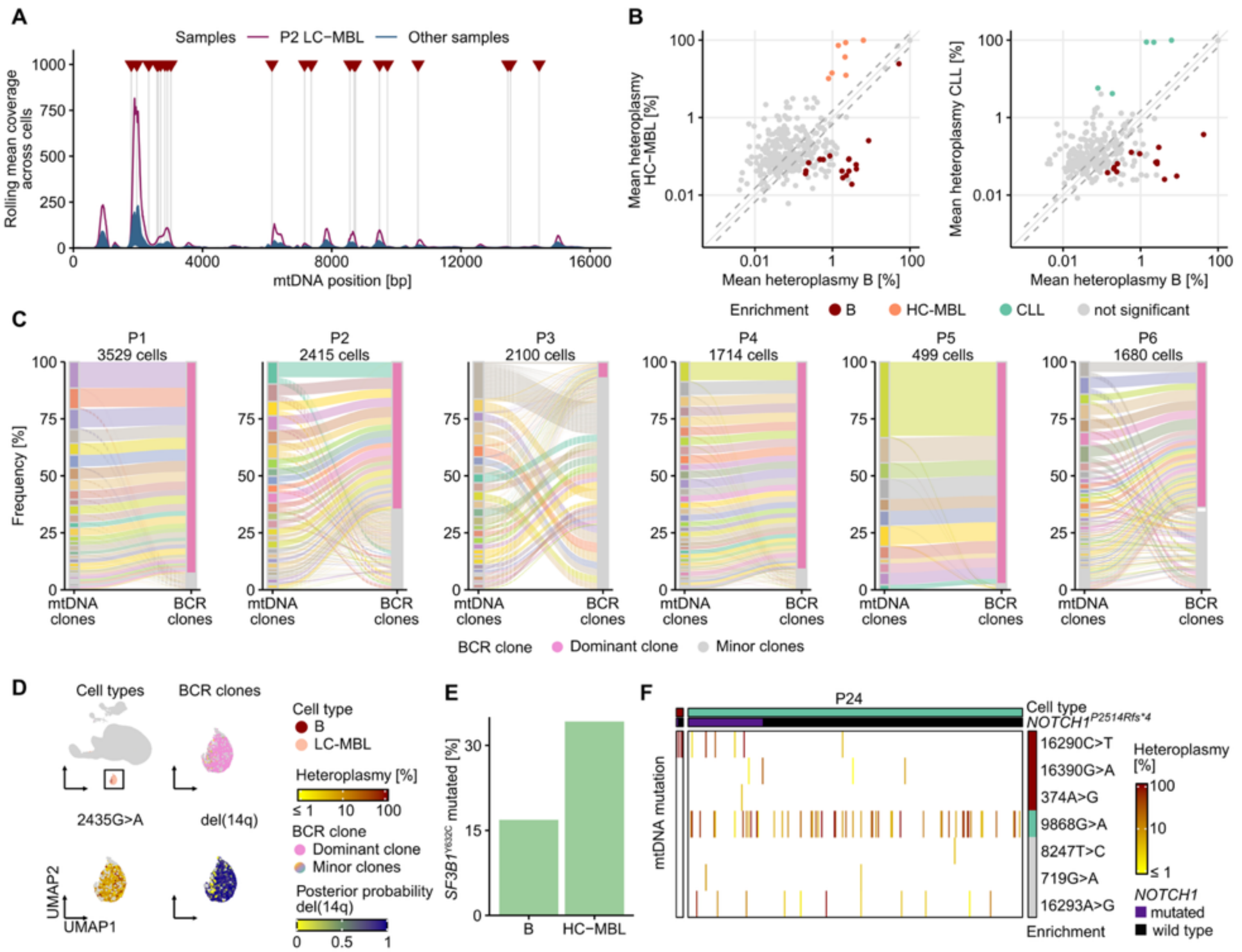

Fig. S14 Identification and tracking of MBL/CLL subclones.

A Enriched mtDNA coverage in P2 (LC-MBL) compared to all other scRNA-seq analysed samples.

B Stability of heteroplasmy changes between physiologic B cells and HC-MBL (left), and CLL, respectively (right). Based on the Wilcoxon rank sum test and Benjamini-Hochberg FDR, ≤ 0.05 in combination with 1.5-fold change threshold (dashed lines).

C Alluvial plot per LC-MBL patient of mtDNA mutation clones' matching with BCR sequence clones based on nanoranger data.

D UMAP representation of cell types, BCR clones, 2435G>A mtDNA mutation, and *del*(14q) in P1.

E Quantification of *SF3B1*^{Y632C} mutated cells in residual physiologic B cells and HC-MBL cells of P12.

F Heatmap displaying the heteroplasmy of mtDNA mutations with significantly changing heteroplasmy between physiologic B cells and CLL cells in P24 and cooccurrence of *NOTCH1*^{P2514Rfs*4} based on mtscATAC-seq data.

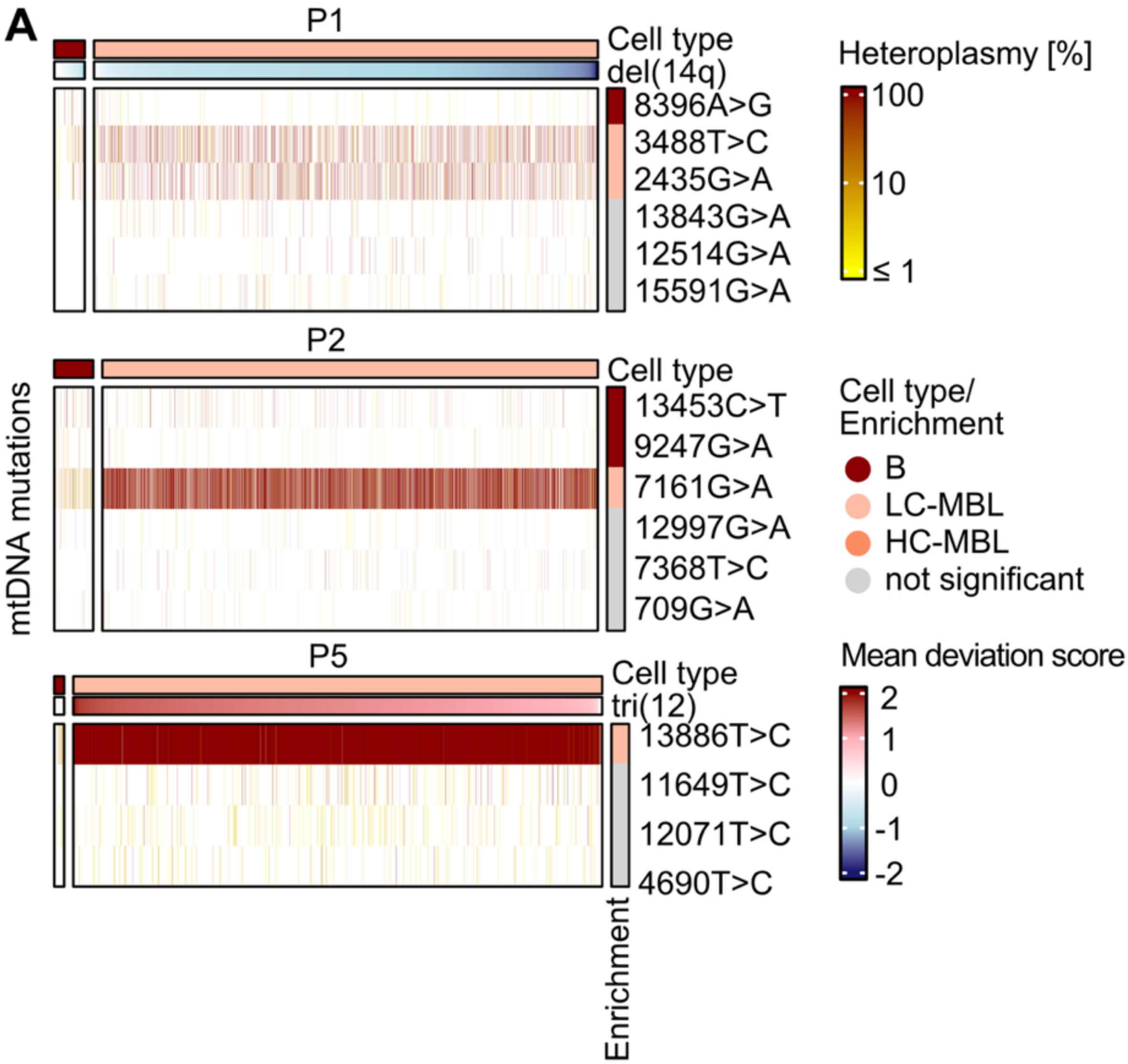

Fig. S15 Mitochondrial DNA (mtDNA) mutation heteroplasmy abundance in physiologic B and LC-MBL cells. **A** Heteroplasmy heatmaps from mtscATAC of mtDNA mutations with significantly changing heteroplasmy between physiologic B cells and LC-MBL cells. Chromosomal aberrations identified from scATAC-seq data are indicated where present.

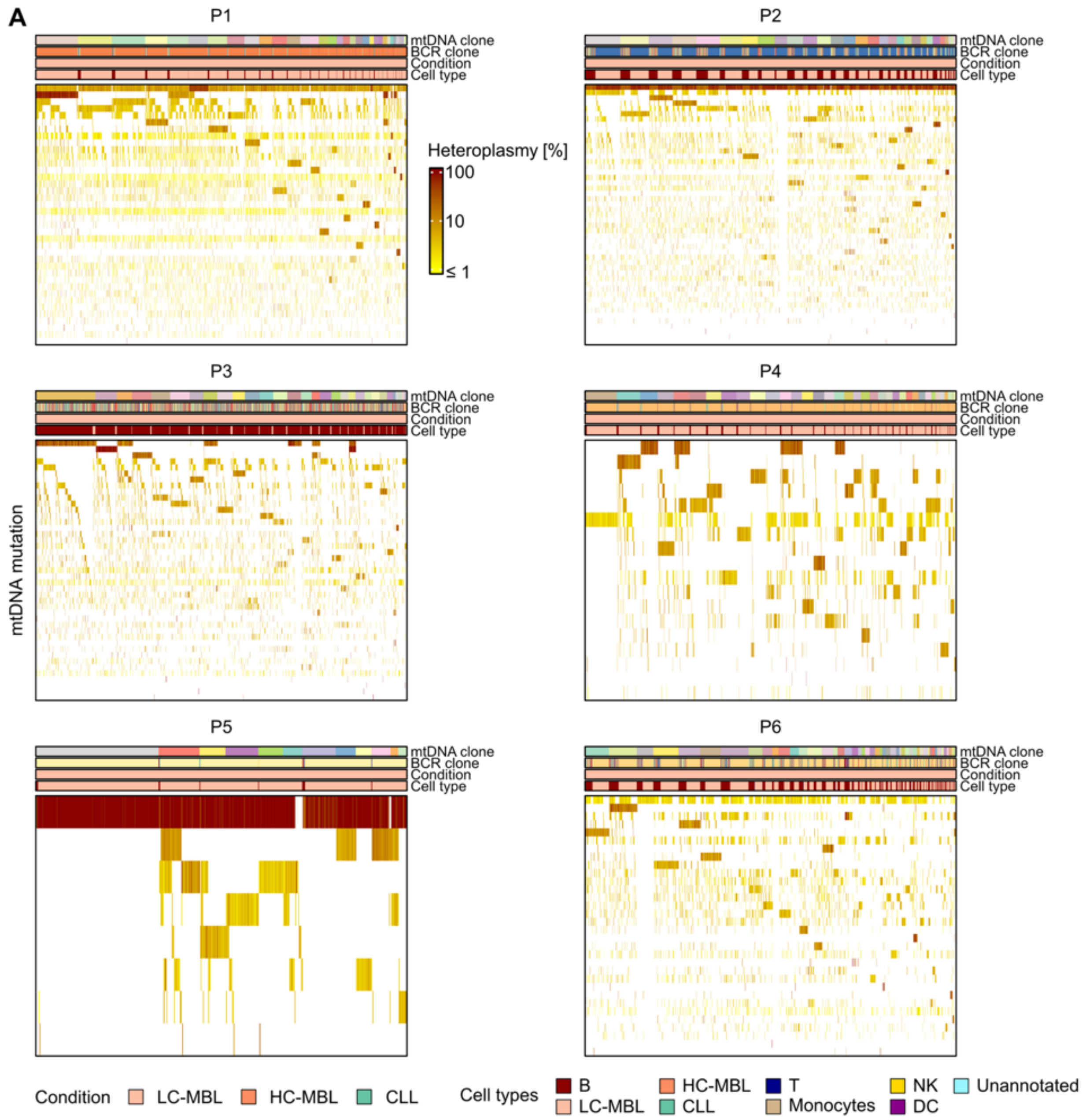

Fig. S16 Mitochondrial DNA (mtDNA) mutation-based clones align with BCR clonotypes.
A High confidence, mtscATAC-seq mtDNA mutations were re-discovered in the mtDNA transcriptome via the nanoranger protocol. For this, mtDNA clones were called based on transcriptomic heteroplasmy and matched with BCR clone annotation for each LC-MBL patient.

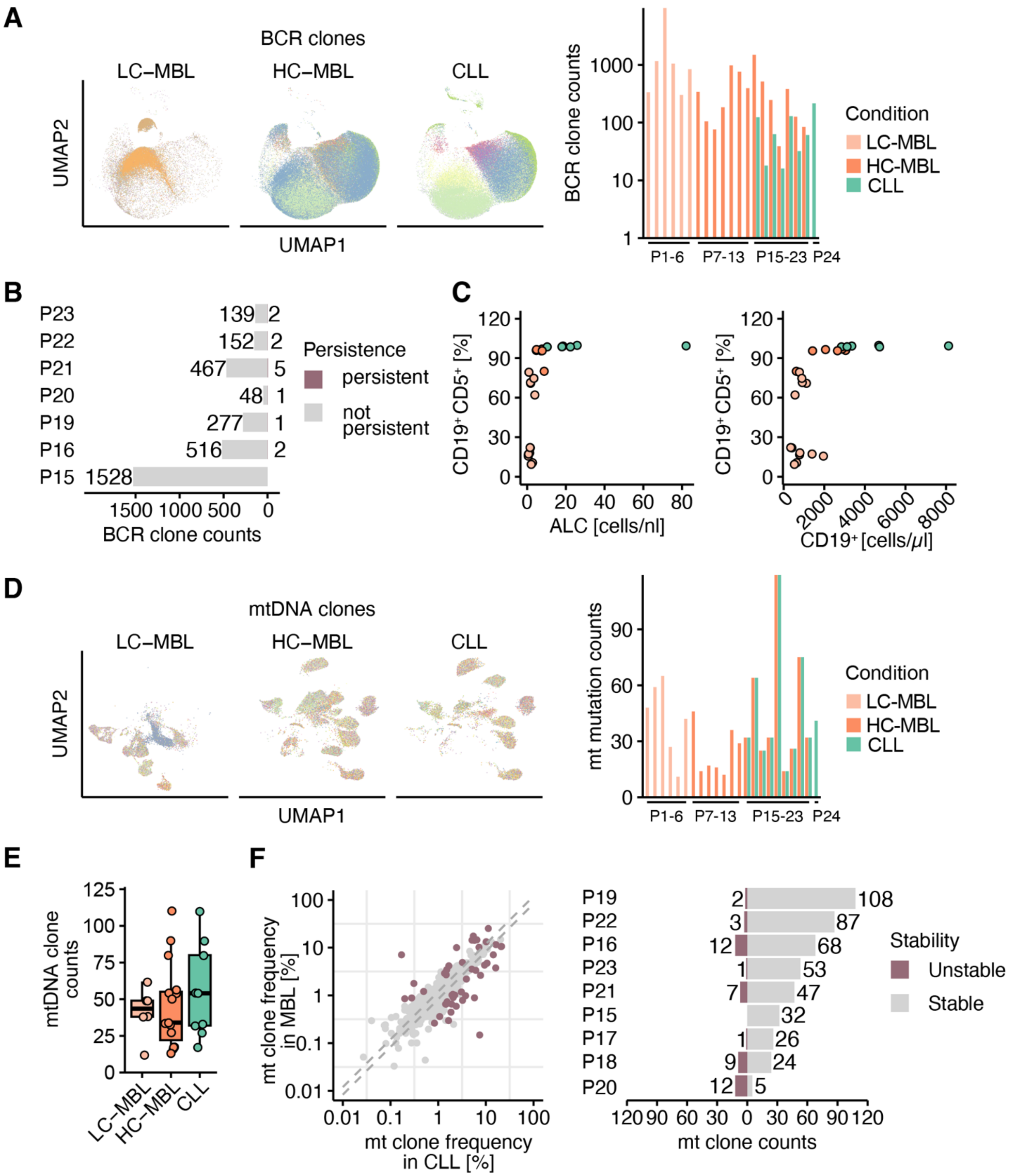
A
BCR clones
LC−MBL
HC−MBL
CLL
UMAP2
UMAP1
BCR clone counts
1000
100
10
1
P1-6
P7-13
P15-23
P24
Condition
LC-MBL
HC-MBL
CLL
B
P23
P22
P21
P20
P19
P16
P15
139
152
467
48
277
516
1528
2
2
5
1
1
2
1500 1000 500 0
BCR clone counts
Persistence
persistent
not persistent
C
CD19⁺CD5⁺ [%]
ALC [cells/nl]
CD19⁺ [cells/µl]
D
mtDNA clones
mt mutation counts
E
mtDNA clone counts
F
mt clone frequency in MBL [%]
mt clone frequency in CLL [%]
P19
P22
P16
P23
P21
P15
P17
P18
P20
2
3
12
1
7
1
9
12
108
87
68
53
47
32
26
24
5
mt clone counts
Stability
Unstable
Stable

Fig. S17 Quantification of MBL/CLL subclonal dynamics.

A UMAP representation of clones detected in LC-MBL (P1-6), HC-MBL (P7-13, P15-23), and CLL (P15-16, P19-24) stages based on scBCR CDR3 sequence (left). Per sample BCR clone counts (right).

B Persistence of BCR clones between patient-matched HC-MBL and CLL samples.

C Flow cytometry quantification of clonal $CD19^{+}$ $CD5^{+}$ cells over absolute lymphocyte count (ALC) (left) and all $CD19^{+}$ cells (right) of 13 LC-MBL cases (P2, P3 and P6 from this cohort and 10 additional LC-MBLs), 6 HC-MBLs and 6 CLL cases.

D UMAPs of subclones detected in LC-MBL (P1-6), HC-MBL (P7-13, P15-23), and CLL (P15-16, P19-24) stages based on mtDNA mutations' heteroplasmy (left). Per sample mtDNA mutation counts (right).

E Number of mtDNA-defined subclones across LC-MBL, HC-MBL and CLL.

F Stability of mtDNA mutation subclone frequency between patient-matched HC-MBL and CLL samples was assessed using a χ^2-test with subsequent Benjamini-Hochberg correction (FDR ≤ 0.05) and an absolute threshold of $\geq$ 1.2-fold change (dashed lines) (left). Quantification of the stability of mtDNA mutation subclones (right).

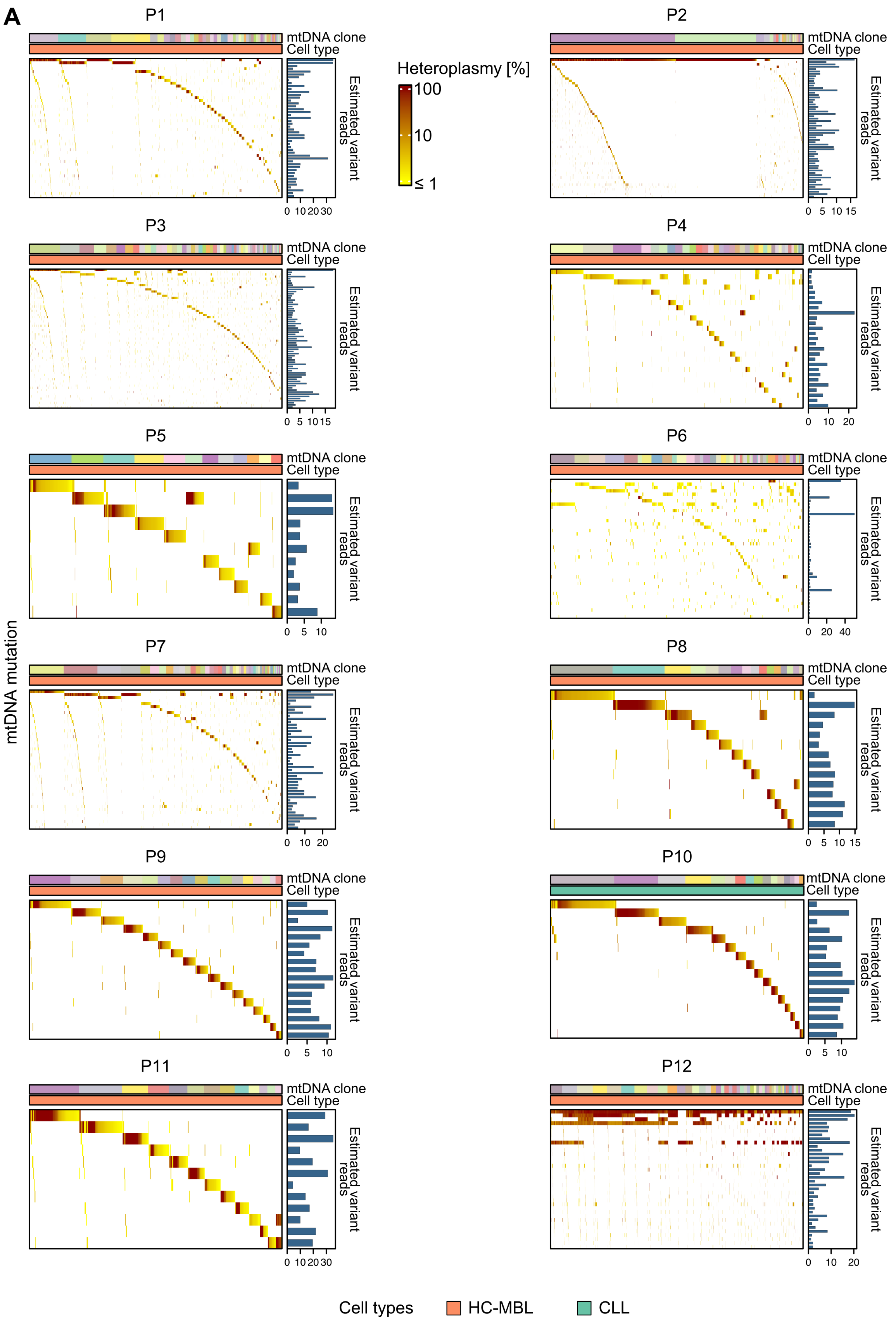
A
P1
P2
P3
P4
P5
P6
P7
P8
P9
P10
P11
P12
mtDNA clone
Cell type
Estimated variant reads
Heteroplasmy [%]
100
10
≤ 1
mtDNA mutation
Cell types
HC-MBL
CLL

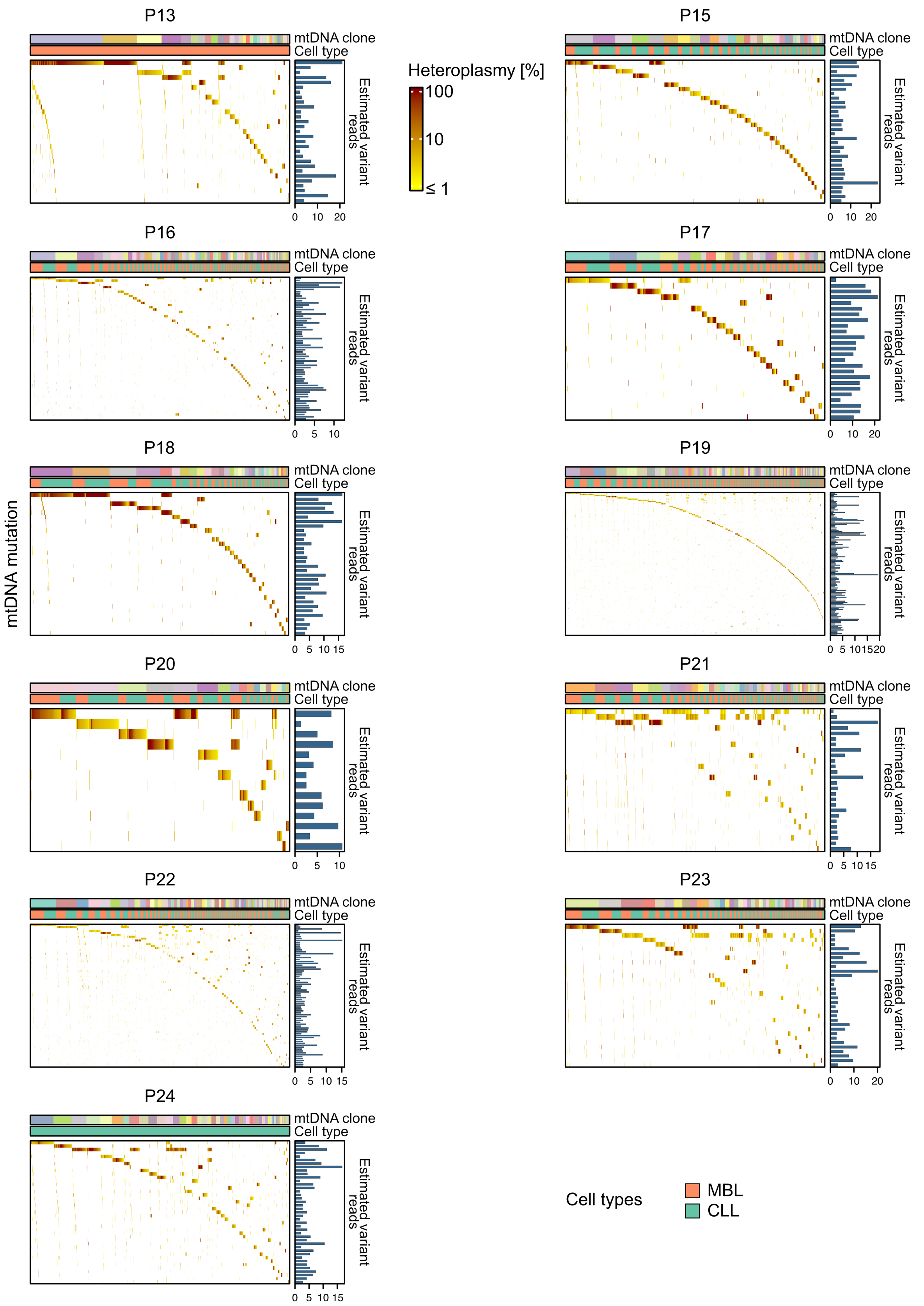
P13
P15
P16
P17
P18
P19
P20
P21
P22
P23
P24
mtDNA clone
Cell type
Estimated variant reads
mtDNA mutation
Heteroplasmy [%]
100
10
≤ 1
Cell types
MBL
CLL

Fig. S18 Mitochondrial DNA (mtDNA) mutation-based clone calling with mtscATAC-seq data.
A mtDNA clones were called based on heteroplasmy of high confidence clonal mtDNA mutations for each patient. Estimated variant read depth was calculated from mean heteroplasmy and mean depth of cells carrying the respective mtDNA mutation.

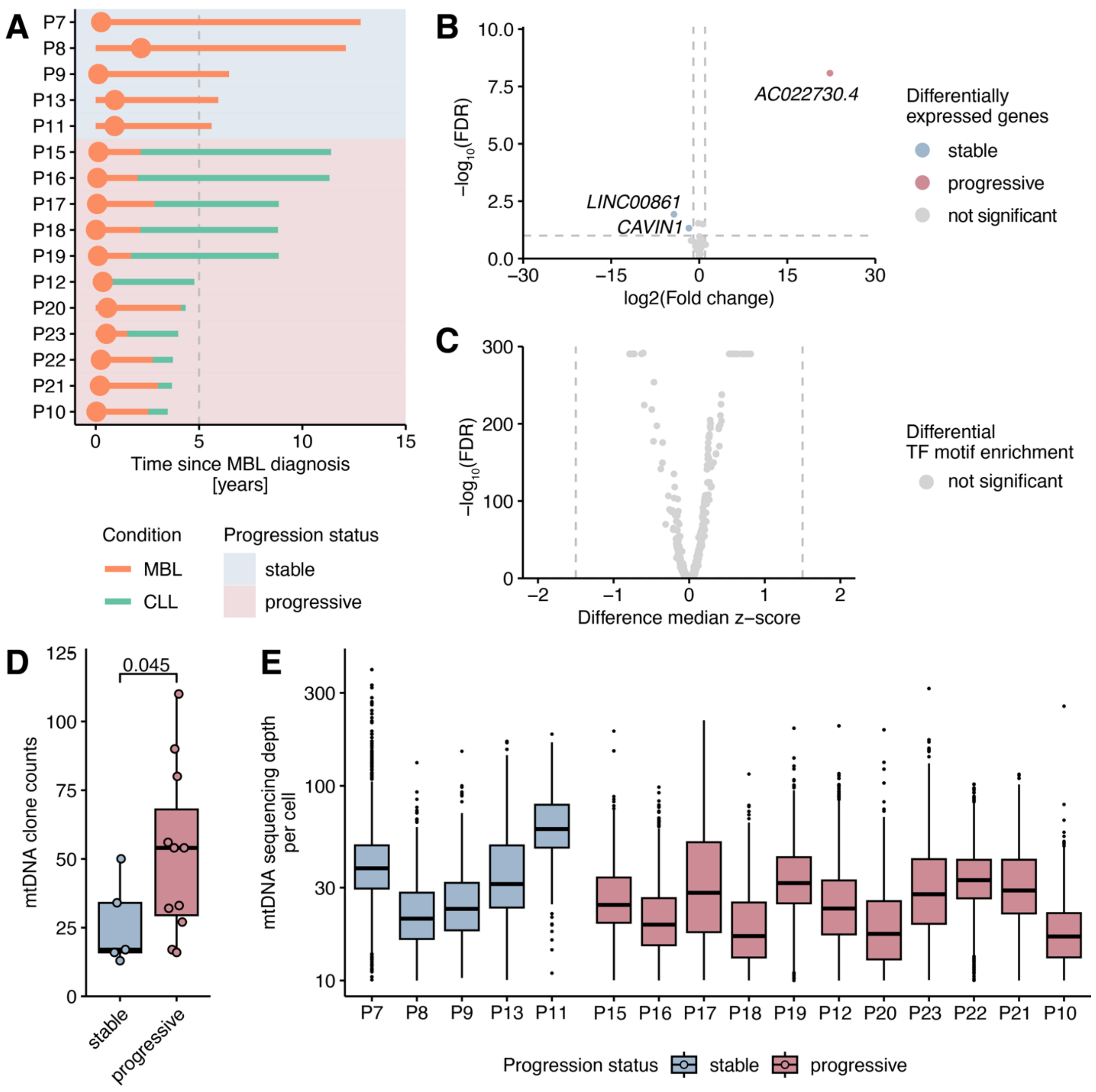

Fig. S19 Mitochondrial DNA mutation (mtDNA)-based clones in stable and progressive HC-MBL.
A Cohort stratification of stable (>5 years) and progressive (<5 years; n=11) HC-MBL cases transitioning to CLL.
B Differential gene expression analysis of stable (n=5) vs. progressive HC-MBL patient samples (n=9) using a Wald test. Dashed lines indicate significant genes with an absolute log2 fold change > 1 and -log10(FDR) > 1.
C Pseudo bulk transcription factor (TF) motif enrichments across stable (n=3) and progressive HC-MBLs (n=6). Z-scores are based on peaks from scATAC-seq data. Statistical testing using Wilcoxon rank sum test (Benjamini-Hochberg correction FDR ≤ 0.05). Dashed lines indicate the threshold of significant changes with an absolute difference of median z-scores > 1.5.
D, E Number of mtDNA clones (D) and mtDNA sequencing depth (E) in stable (n=5) and progressive HC-MBL (n=11). Statistical testing with Student t-test.
